# A community conversation process to establish resident and service provider perspectives on needs related to use and treatment of opioids and substances

**DOI:** 10.3389/fpubh.2025.1678130

**Published:** 2026-01-27

**Authors:** Jennifer Shepheard, Thomas Mundy, Ashlie Watts, Jana Pushkin, LeTeisha Gordon, Patrice Shelton, Courtney Blondino, Katherine Werner, Elizabeth Prom-Wormley, Melissa Viray

**Affiliations:** 1 Richmond & Henrico Health Districts, Virginia Department of Health, Richmond, VA, United States; 2 Department of Epidemiology, Virginia Commonwealth University, Richmond, VA, United States; 3 Finding Redemption Through Enlightening and Education, Richmond, VA, United States; 4 A Better Day Than Yesterday, Richmond, VA, United States; 5 Department of Health Studies, University of Richmond, Richmond, VA, United States

**Keywords:** community based participatory research, community-engaged research, opioids, resource needs, substance use

## Abstract

**Introduction:**

Rates of fatal overdose due to opioid and substance use in Richmond, Virginia increased from 44.6 in 2018 to 129.5 per 100,000 city residents in 2023. The underlying contexts surrounding the increase in substance use and overdoses in Richmond, Virginia remains poorly understood.

**Methods:**

Using community based participatory research principles (CBPR), a series of “community conversations” with neighborhood residents were conducted between May–December 2023. These events included educational information, resource connection, and facilitated qualitative focus group discussion on factors contributing to substance use and overdose, as well as resource needs of people engaged in substance use. Participants also completed a survey on personal substance use experience.

**Results:**

Approximately 121 adults participated in 11 community conversations. Of 107 participants with survey data, 37.4% and 47.4% reported ever engaging in non-prescription or prescription opioid use, respectively. Factors leading to local overdose reflected three themes: (1) Diversity in Substance Use Narratives, (2) Coping with Impactful Life Events and Mental Health Experiences, and (3) Community- and Institutional-level Access to Substances. Resource needs were categorized as three themes: (1) Knowledge and Information-sharing around Substance Use, (2) Community Cohesion and Social Support, and (3) Consistent Wraparound Resource Support.

**Discussion:**

Richmond-area resident perspectives align with results from prior studies while highlighting locally-nuanced factors regarding prevention, treatment, and community supports. Participants emphasized the need for comprehensive, multi-pronged approaches that expand clinical and corrections-based services, improve resource navigation, and provide personalized, family-engaged support to strengthen neighborhood cohesion. These insights showcase the value of CBPR in elevating lived experience to guide actionable, community-tailored strategies.

## 1 Introduction

In 2022, the Richmond and Henrico Health Districts (RHHD, the local health district serving the City of Richmond) began development toward a process that integrates surveillance data and community perspectives with a goal of reducing the impact of drivers of overdose in Richmond, Virginia. This work was conducted in response to prior increases in overdoses due to opioid and substance use. For example, the rate of fatal drug overdoses in 2023 for Richmond (129.5 per 100,000 city residents) was over four times greater than the overall rate for the state (30.5 per 100,000 state residents). Further, the rate of fatal opioid overdoses among non-Hispanic Black residents nearly quintupled from 38.1 per 100,000 in 2018 to 179.8 per 100,000 in 2023 (1).

The increase in overdose rates encouraged a collaborative partnership to plan and implement a project that identified the factors contributing to the patterns of local substance use and overdose surveillance data. This project used qualitative methods to conduct and analyze a series of community conversations, applying community-based participatory research (CBPR) (2–5) principles throughout all stages (Table 1). There were three major groups of partners in this collaboration, each with specific strengths. RHHD had access to the most current surveillance data, a comprehensive network of community health workers supporting established relationships with community residents, a dedicated staff to engage in several project coordination tasks (e.g., project management, data management, conducting focus groups, and formal public relations/advertising development). The local community organizations (A Better Day Than Yesterday and Finding Redemption through Enlightening and Education) previously partnered with RHHD and maintained strong relationships with the substance use community, including people engaged in substance use, those in recovery, and recovery-focused service providers. The academic partner (Virginia Commonwealth University) had prior experience in conducting and engaging in rapid analysis of large-scale, community-engaged research projects to encourage equitable and efficient project development, results dissemination, and program implementation (Table 2).

**Table 1:** Application of CBPR principles in study development and implementation.

**CBPR principle**	**Community conversation application**
1. Recognize the community as a unit of identity	Included cross-sector partnerships between community organizations, community health workers, academic partners and local health department. Relationship building and trust between partners was built upon throughout the process.
2. Build on strength and resources within the community	Established Community Health Workers located in specific neighborhoods served as trusted individuals to recruit participants and host conversations in partnering community spaces.
3. Facilitate collaborative, equitable partnership in all phases of the research	Community Organizations and Community Health Workers advised and provided feedback to the local health department and academic researchers from the initial grant application to data collection to the manuscript drafts.
4. Promote co-learning and capacity building among all partners	The academic partner advised the local health department and community organizations on the CBPR principles and conducting community research. Co-learning and capacity building occurred across partners on the research process, facilitation of focus groups, data collection and analysis of qualitative data.
5. Integrate and achieve a balance between research and action for the mutual benefit of all partners	Modifying the approach from recommendations of community partners to include educational sessions at each focus group to ensure each individual gained knowledge and benefitted from the focus groups.
6. Emphasize local relevance of public health problems and ecological perspectives that recognize and attend to the multiple determinants of health and disease	The study was designed based on surveillance data of overdoses in Richmond. The study focused on examining the multiple perspectives, including the impact of local contextual factors, and social determinants of health contributing to the population rates of overdose.
7. Involve systems development through a cyclical and iterative process	Utilized routine post-event and bi-weekly meetings to allow for feedback, examination of successes and troubleshooting limitations.
8. Disseminate findings and knowledge gained to all partners and involving all partners in the dissemination process	Early results were shared with community partners and other interested parties. Additionally, results from the prior 9 conversations were shared at the last two conversations with conversation participants. Full results have been shared with community members, community stakeholders, and other public health professionals.
9. Establish a long-term commitment to the process	Study was designed using joint CBPR, community needs assessment, and epidemiology principles to understand the current drivers of substance use and overdose. Immediate action and long-term strategies are being developed among local health department, academic partners, and community stakeholders.

**Table 2:** Partners and their roles.

**Category**	**Partner**	**Role**
Local government	Richmond and Henrico Health Districts (RHHD) staff (population health epidemiologists, health equity specialist, substance use coordinator, community health workers, deputy health director)	Led the team through the study design process, data collection, analysis, interpretation of results, presentation, and dissemination of results
Academic partner	Virginia Commonwealth University (VCU)	Advised on statistical analysis, study design, qualitative analysis, interpretation of results
Community organizations	A Better Day Than Yesterday; Finding Redemption through Enlightening Education (FREE)	Recruited participants and supported the logistical planning for the community conversations, interpretation, and presentation of results
Funding agency	National Association of County and City Health Officials (NACCHO)	Provided funding, technical assistance, and peer learning

This article details areas where CBPR principles (2–5) were incorporated to support effective partnership by (1) describing the processes of study design, data collection, and data analysis related to community conversations; (2) summarizing stages in the development and execution of community conversations that supported effective project partnership; and (3) detailing results and lessons learned for translation into actions that will support resident substance use needs and guide future legislative decision-making or program planning.

## 2 Methods

**Figure 1:**
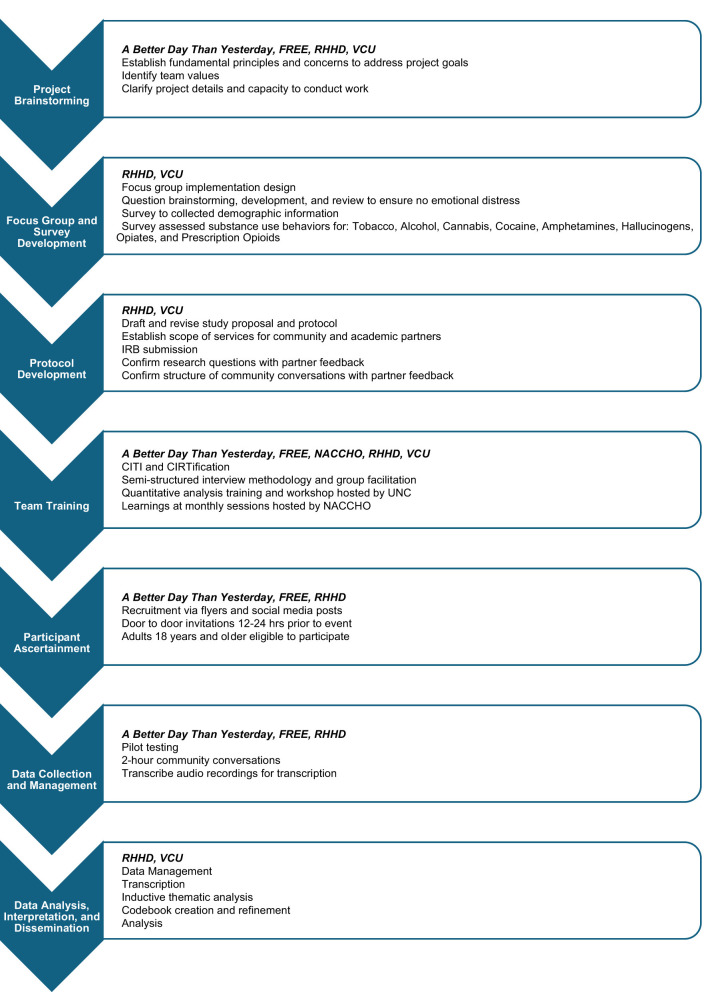
Project stages.

Community conversation development was guided by two research questions: (1) What factors contribute to the increase in overdoses in Richmond, and (2) What community-facing resources and support services are missing that could be implemented? The project also sought to identify barriers to accessing community resources related to opioid overdose. Institutional Review Boards within the Virginia Department of Health (VDH Study #70060) and Virginia Commonwealth University (HM20027269) approved the study protocol. Project stages are highlighted in Figure 1.

### 2.1 Community conversation development

#### 2.1.1 Project brainstorming

The project began in October 2022. Seven brainstorming meetings were conducted between November 2022 and February 2023 to establish fundamental principles for addressing project goals. Three team values were identified: (1) Consulting regularly with community partners to produce a study design and events that would resonate with community residents; (2) Producing events that offer an immediate (non-financial) benefit to participants; and (3) Establishing non-judgmental and supportive spaces for all participants to openly share thoughts. This phase also clarified project details and partner capacity to conduct work.

#### 2.1.2 Focus group development

The focus group protocol was created iteratively across two stages based on guiding principles and concerns identified during brainstorming sessions. The first stage developed the logistics to implement community conversations. The second stage identified a set of potential community conversation questions (Appendix Table A.1). Additionally, contributors reviewed question wordings to incorporate language familiar to participants and ensure low risk for causing emotional distress.

#### 2.1.3 Survey development

A self-report, paper-pencil survey was developed to collect demographic and substance use information that participants may not feel comfortable disclosing in group discussion. Survey items were compiled from PhenX and ASSIST-Lite (6–9). Survey development occurred from December 2022 to July 2023.

#### 2.1.4 Training

All core research team members engaged in training of ethical conduct of research either through the Collaborative Institutional Training Initiative (CITI; RHHD and VCU) or Community Involvement in Research Training (CIRTification; community partners and RHHD staff working with community residents) (10).

Community partners and RHHD team members who served as facilitators received facilitation training to practice IRB-approved semi-structured methods for conducting community conversations. RHHD team members who served as a project lead or conducted data analysis received technical training through NACCHO and from another health department on substance use topics (e.g., stigma, harm reduction) as well as on community engagement. Training ensured culturally sensitive engagement with community partners and participants.

### 2.2 Pilot testing

A pilot event was conducted to test aspects of the community conversation process, including: (1) recruitment strategy, (2) timing and activity length, (3) participant engagement, and (4) audio recording setup. A convenience sample of 14 individuals participated. The pilot discussion lasted approximately 2 hour. Results demonstrated the need for additional time to encourage greater depth in responses, and community conversations were extended from 1 hour to 1.5 hours.

### 2.3 Participant recruitment

Community partners and community-facing RHHD team members conducted recruitment by creating event advertisements and posting them to social media (e.g., Instagram). Community health workers, community partner programs, local treatment centers, and recovery houses distributed flyers. Many community conversations were located in neighborhood resource centers maintained by RHHD. RHHD Community Health Workers, health equity specialists, and community partners conducted door-to-door invitations 12–24 hours before an event. Community partners coordinated other conversation sites in their service area or in locations where they had a relationship with community residents. Adults ages 18 and over were eligible to participate.

### 2.4 Data collection

Community conversations were held during a 2-hour time frame on weekday evenings. Community conversations consisted of four activities: an educational presentation, informed consent, focus group discussion, and survey completion. During the final two conversations, a results dissemination component replaced focus group discussion. Upon arrival, individuals received: a resource connection request form, a guide to the RHHD community health worker program, a brochure of local substance use support services, consent information, and the substance use behavior survey (Appendix Figures B.1–5). The first 30 min of each conversation began with a shared meal and an educational presentation. Educational topics ranged from naloxone training to career training opportunities, based on input from the community partner as well as feedback during the pilot testing phase. After the educational presentation, informed consent was obtained prior to starting the focus group conversations. This format allowed for individuals to be able to participate in the education and meal and choose whether to participate in the focus group. Conversations were audio recorded and 4–6 notetakers ensured content fidelity. A written response option was available for participants who did not wish to participate verbally. Upon completion of the focus group and survey, all participants received a $25 gift card.

### 2.5 Data management

Audio recordings and digital notes (compiled from hard copy notetaker observations and written responses) were used to produce focus group transcripts. Anonymized transcripts themselves were created using Microsoft Word's automatic transcription function, followed by manual editing with hard copy notes.

Survey data were collected as paper-and-pencil responses and de-identified in accordance with Virginia Department of Health Information Security and Confidentiality policies prior to manual electronic entry. Digitized data were securely stored on encrypted laptops for further analysis.

### 2.6 Data analysis and interpretation

Summary statistics were estimated using frequency counts and proportions as well as means and variances in R, Version 4.1.2 (“Bird Hippie”) (11).

A four-step thematic content approach was adapted from various qualitative analysis processes described in the literature (12). In Step 1 (“Data Exploration”) the team used mind mapping to generate preliminary codes based on key ideas from the conversation data (JS, JP), and reviewed them to ensure neutral language and minimize redundancy (EPW, CB). Step 2 (“Systematic Code Identification”) involved independently open-coding the conversation data by hand to assess whether preliminary codes adequately captured the content and sentiment expressed in the data (JS, JP). In Step 3 (“Codebook Refinement”), the team developed a comprehensive codebook, incorporating new codes that emerged in Step 2 (JP) and further revising content and structure (JS). Two additional rounds of review confirmed the final codebook's organization and ensured that all terminology reflected standards for inclusive language (Appendix Table C.1). In Step 4 (“Digital Coding”), transcripts and notes were digitally coded according to the final codebook using NVivo Version 14 (13) (JP) and was reviewed for accuracy and consistency (JS).

## 3 Results

### 3.1 Descriptive statistics

A total of 121 unique participants attended 11 community conversation events from May to December 2023 (*N* = 4–18 participants per conversation). Of all participants, 107 completed a survey (88.4%). Approximately 40% of participants did not provide data on race, gender, and zip code demographic variables (Table 3).

**Table 3:** Distributions of demographic characteristics.

**Variable**	**Total participants (*N* = 107)**
Mean age in years (*SD*)	40.2 (12.4)
**Age group**	
18–24	7 (6.54%)
25–29	12 (11.22%)
30–39	39 (36.45%)
40–49	20 (18.69%)
50–59	11 (10.3%)
60+	11 (10.3%)
Missing, not reported	6.54%
**Race/ethnicity**	
American indigenous or Alaskan Native	3 (2.80%)
Black or African American	48 (44.86%)
Hispanic or Latine	4 (3.74%)
Two or more Races	2 (1.87%)
White	7 (6.54%)
Missing, not collected	39.25%
Missing, not reported	0.94%
**Gender** ^*^	
Female	32 (29.91%)
Male	17 (15.89%)
Missing, not collected	40.19%
Missing, not reported	14.02%
**Richmond city resident** ^**^	
Yes	53 (49.53%)
No	8 (7.48%)
Missing, not collected	39.25%
Missing, not reported	3.74%

^*^Additional gender identity options (i.e., Transgender Woman, Transgender Man, Non-Binary, Agender, Gender Not Listed) were included in the survey but not endorsed in the study sample.

^**^Richmond City resident status assessed by zip code.

The prevalence of lifetime substance use was highest for alcohol (88.4%), nicotine (81.4%), and cannabis (78.8%). Nearly half of participants reported ever using prescription opiates (47.4%) or cocaine (46.9%), and 37.4% reported ever using non-prescription opiates. On average, the age of initiation for alcohol, cannabis, hallucinogens, and prescription opioids was between 15.5–17.8 years old and 20.2–24.0 years old for amphetamines/methamphetamines, cocaine, nicotine, and non-prescription opiates. Across substances, at least two-thirds of participants reporting use also reported one or more quit attempts. Additionally, compared to other substances, a higher percentage of participants who had ever used cocaine, nicotine, non-prescription opiates, or prescription opioids reported concern from a relative, friend, or health professional regarding their use (Table 4).

**Table 4:** Distributions of substance use behaviors.

**Substance used**	**Age of initiation**		**Lifetime initiation**		**Current use** ^*^		**Quit attempt**		**Concern** ^*^	
**Mean**	**SD**	***N***	**%**	***N***	**%**	***N***	**%**	***N***	**%**
**Alcohol**										
No	16.6	4.9	12	11.65	40	43.96	25	32.05	48	62.34
Yes			91	88.35	51	56.04	53	67.95	29	37.66
Total			103	100	91	100	78	100	77	100
**Amphetamines**										
No	20.2	5.0	57	57.58	35	83.33	10	27.03	26	70.27
Yes			42	42.42	7	16.67	27	72.97	11	29.73
Total			99	100	42	100	37	100	37	100
**Cannabis**										
No	15.6	5.4	21	21.21	46	58.97	20	30.77	44	72.13
Yes			78	78.79	32	41.03	45	69.23	17	27.87
Total			99	100	78	100	65	100	61	100
**Cocaine**										
No	21.1	6.4	52	53.06	35	76.09	4	10.26	19	48.72
Yes			46	46.94	11	23.91	35	89.74	20	51.28
Total			98	100	46	100	39	100	39	100
**Hallucinogens**										
No	17.8	4.6	64	66.67	29	90.62	8	30.77	23	85.19
Yes			32	33.33	3	9.38	18	69.23	4	14.81
Total			96	100	32	100	26	100	27	100
**Nicotine**										
No	24.0	8.2	19	18.63	27	32.53	16	21.62	38	54.29
Yes			83	81.37	56	67.47	58	78.38	32	45.71
Total			102	100	83	100	74	100	70	100
**Opiates**										
No	22.9	9.9	62	62.63	28	75.68	5	15.62	16	50
Yes			37	37.37	9	24.32	27	84.38	16	50
Total			99	100	37	100	32	100	32	100
**Prescription opioids**										
No	15.5	6.2	51	52.58	33	71.74	7	19.44	21	60
Yes			46	47.42	13	28.26	28	77.78	14	40
Total			97	100	46	100	36	97.22	35	100

^*^Current use- Past 3-month use; Concern- Relative, friend, or health professional expressed concern about use or suggested reduction.

### 3.2 Conversation findings: “What factors are contributing to the increase in overdoses in Richmond?”

Three thematic areas were identified as contributing to the increase in overdoses in Richmond: (1) Diversity in Substance Use Narratives, (2) Coping with Impactful Life Events and Experiences, and (3) Community- and Institutional-level Access to Substances. In aggregate, these themes emphasized a variety of life experiences resulting from several factors and the need to account for such diversity in future planning (Appendix Table D.1). The first two themes described factors that contribute to increases in substance use and were also considered to partially intersect with those influencing increases in overdose. In particular, the effect of stressful life events and emotional strains could be experienced uniquely by an individual. Further, the influence of these factors had the potential to compound over time. These highlighted factors were thought to increase the risk for substance use and overdose as well as contribute to the barriers to successful recovery engagement. Prior studies have identified similar themes, shaping the diverse substance use experiences and coping strategies prior to and during the COVID-19 pandemic (14, 15). Given the consistency of the first two themes with results from other studies (14–17), we focus on the Community- and Institutional-level Access to Substances theme and describe their nuances within the Richmond context. The other two themes are detailed in Appendix D.

#### 3.2.1 Community- and institutional-level access to substances

This theme represents two connected perspectives around community substance use access, as perceived by participants. The first perspective highlights the role of community-level behaviors in normalizing patterns of substance use which, in conjunction with factors listed in other themes, also increased the likelihood of use and overdose. Further, participants noted a societal desensitization to overdose and substance use-related death, which they associated with broader community acceptance of these outcomes and their risks. Despite participant conclusion that substance use is common, there was also discomfort in openly discussing the landscape of these behaviors. Participants felt that this lack of open discussion perpetuated overdoses. Nevertheless, such transparency was determined as necessary for community-level change.

##### 3.2.1.1 Normalized patterns of community-level substance use

Across conversations, participants considered substance use to be commonplace, noting its high visibility in Richmond:

“…*You've seen it [substance use] growing up in your whole life. … Everybody who rides across this city, downtown, everywhere that you gotta go, you get a chance to see it with your own eyes before you even indulge at all for your own self.”*

Thirteen participants also identified the role of peer groups in encouraging use, contributing to the perspective of substance use as a normalized behavior:

“*You are being accepted by a whole group of people… because if you don't smoke weed you can't hang around people who smoke weed. They don't wanna be bothered with you*.”

Four participants also identified broad public desensitization to the risks and potential consequences of substance use:

“*Yeah, it used to be– You can see like if someone pass away [from overdose], and you like ‘nah I ain't messing with that stuff.' Now they‘re just like, 'oh, well, yeah, I ain't going to the same person so I‘ll be alright.' It's still ignorant. Now you jump from this person to this person… even though you watched them pass away. But that's what you know, so you go for what you know*.”

##### 3.2.1.2 Widespread access to substances

Participants identified the presence of community-level distributors as a factor that contributed to substance use and overdose. The quality, formulation, or potency of substances could not be guaranteed in any location, which they felt contributed to overdoses.

In addition to distributors based out of personal residences, two types of venues were identified which offered low-cost public access to substances: (1) Pop-up shops advertised via social media.

“*They got pop-ups every day of the week… all around Richmond. They sell ‘shrooms, they sell weed, they sell shatter, they sell wax… Instagram, that's where you mostly find them at*.”

and (2) Corner stores

“*I know at least three corner stores that I could walk into and get an ounce of weed—a pound of weed.”*.

Further, they simultaneously denounced the presence of “drug money” (i.e., money obtained through illegal substances transfers/dealing) and acknowledged the role of this enterprise in keeping community economies functioning:

“*We have a whole hidden economy… it's plaguing our community.” … “That hidden economy is feeding some families. That hidden economy is paying rent.”*

Consequently, participants were conflicted. They could see the act of providing access to substances having the potential to harm others while also serving as a means of economic survival.

Participants also identified three institutional-level venues through which substances could be accessed, despite expectations of strong regulation or oversight: (1) Inside prisons.

“*I got a jail call not too long ago over the phone from a friend of mine… and he was telling me how somebody OD'd in the prison. I didn't even know how that's possible. … They doing drugs, getting them, and the police can't stop them.”;*

(2) Physicians and hospitals who prescribe strong pain medications without adequate monitoring

“*My cousin had a serious back injury. The doctor put him on opiates… three months later, he was hooked on them.”*;

and (3) Substance use treatment programs

“*[The treatment program]… let him get a phone and call all the drug dealers in 30 minutes, and my mama said she'd keep sending money, so he'd keep using… make it so that [drugs] aren't so accessible”*.

##### 3.2.1.3 Challenges engaging in community-level discussions on substance use

Seven participants discussed community-level discomfort when talking openly (i.e., without fear of judgment or stigma) about substance use and associated concepts like mental health and homelessness:

“*We talk about food deserts all day, but we're not talking about this [substance use and drug access].”*

One participant added that lack of open conversation perpetuated misconceptions about the populations affected by substance use in Richmond:

“*It's not just the African American communities! We hear about [substance use] in African American communities… but really, it's happening in Caucasian households, Hispanic households… we just don't know because it's such a thing where it's not talked about.”*

In response, seven participants asserted that a “culture of vulnerability” should be cultivated to build comfort, resilience and solidarity in discussing these issues. They also noted a lack of coordinated community-level action as a contributor to overdose and substance use:

“*Between the mental health and the addiction, ain't nothing gonna change unless these [conversations] keep happening*.”

However, three participants cautioned that conversations rarely translate into tangible actions or results. They attributed this to both a lack of involvement from decision-makers and a lack of confidence among community members in having their voices heard:

“*No one is talking to the very people being impacted… they don't feel they have a voice.”*

### 3.3 Conversation findings: what resources or support services are missing that could be implemented in the community?

Three major thematic areas were identified with respect to resource and support service needs: (1) Knowledge and Information-Sharing Around Substance Use, (2) Community Cohesion and Social Support, (3) Consistent Wrap-Around Resource Support. Additionally, participants identified 13 separate resource categories, representing four major areas of support (Table 5). Despite Richmond's existing wrap-around and one-stop shop resources, responses emphasized the need for simpler navigation and greater resources. The need for wrap around and one-stop-shop resources has consistently been identified in prior studies (18–20). Therefore, we will focus on the themes of Knowledge and Information-Sharing and Community Cohesion and Social Support as they are shaped in distinct ways by Richmond's local history and context. The remaining theme is described in detail in Appendix D.

**Table 5:** Community resource requests by category (*N* = 164).

**Category**	**Summary requests**	***N* (%)**
Housing resources	•Affordable and second-chance housing •Neighborhood and shelter safety •Expanded shelter space with extended hours	25 (15.2%)
Substance use treatment and harm reduction	•Affordable Residential Treatment Facilities •Medical support during detoxification •Treatment as an alternative to incarceration •Continued “aftercare” post-treatment •Integrated harm reduction and prevention efforts	22 (13.4%)
Employment and financial resources	•Age-inclusive and second-chance job opportunities •Job search help and workforce development •Personal finance and money management services	19 (11.6%)
Mental and emotional health	•Accessible mental health care for youth, veterans, etc. •Resources for establishing adaptive coping mechanisms •Crisis care without police intervention	17 (10.4%)
Social support	•Peer recovery support groups •Wraparound support for the family unit •Faith-based community creation	16 (9.8%)
Education	•Effective substance use messaging (e.g., PSAs) •Transparent community data sharing •Resources for independent learning	13 (7.9%)
Community gathering space	•Refurbished and revitalized community centers •Safe outdoor play spaces for kids •Family-focused community events	11 (6.7%)
Food resources	•Free or low-cost community meals •Food assistance in neighborhoods and shelters	9 (5.5%)
Sports and recreation	•Sports, camps, and activities for kids •Access to parks and recreation •Police athletic league for youth relationship building	9 (5.5%)
Transportation	•Transportation to treatment and service centers •Transportation for children to extracurricular activities •Mobile resource vans	7 (4.3%)
Communication	•Community voice and self-advocacy training •Relevant neighborhood guest speakers	6 (3.7%)
Support for resource providers	•Funding for existing community programs •Professional development for care providers	6 (3.7%)
Clinical health resources	•Community health fairs •Mobile preventive care screenings •Medical care for older adults	4 (2.4%)

#### 3.3.1 Knowledge and information-sharing around substance use

This theme offers a background on how participants learned about substance use, as well as the topics needed to address knowledge gaps. Social media was identified as a major source of information. Further, inconsistency of content quality was identified as a concern. However, participants also identified educational topics and messaging strategies that could have community-level impact.

##### 3.3.1.1 Social media

Fifteen participants mentioned social media as a source of multiple types of substance-related information. First, media content was identified as a source of positive perceptions around particular substance use patterns. One participant noted the popularity of media-transmitted substance use messages:

“*‘I knew the perc was fake, but I still ate it because I'm a gremlin.' That's probably the most recited line of that whole rhyme [from a song]”*.

Participants considered this type of content, in addition to the substance use attitudes and behaviors modeled by other adults in their lives, as acceptance of this pattern of use. Second, social media was used to access knowledge-based content on substance trends like “gas station heroin,” carfentanyl, and xylazine. Third, social media was used to connect with counseling-related resources related to substance use. One service provider noted,

“…* we do therapy talk Wednesdays, we do it in a public setting because what we do know is everybody links to social media*.”

Fourth, connections with public health educational campaigns were occurring through social media. However, their impact was mixed. Four participants were disappointed with the lack of commercials and messaging against substance use because they found such advertisements to be memorable. In contrast, two said they were ineffective or encouraged experimentation.

##### 3.3.1.2 Community education and awareness

There were four areas for which participants wanted information. First, participants wanted to understand the relationship between substance use and mental health:

“*I feel that mental health is a key contributor*—*people aren't as informed about mental health and the effects of it… especially men. They feel embarrassed. Stronger mental health community. Eliminate the stigma*.”

Second, identifying effective coping strategies to manage stressors and trauma—thereby reducing the likelihood of substance use—was considered a critical skill, particularly for children:

“*So… all of the stresses that these young people or these youth are growing up with- It's hard to want to say that an 8-year-old is using, but…, what if that's what the 8-year-old is using to cope to get through seeing that dead body, getting on the school bus, and actually getting to school?*”

Third, participants requested information on safer substance use practices, including the identification of products that might be mixed with other substances. In particular, participants requested information on preventing overdose through training on naloxone administration. Six participants had never heard of, had access to, or used naloxone before, but wanted to learn how to avoid overdose death in their communities.

Fourth, 12 participants wanted to understand substances' physiological effects to recognize adverse reactions and sensations that could signal potential harm or precede overdose:

“*All types of stuff fighting more easily against each other, so then that might be giving the body a different*—*like an adverse effect*.”

#### 3.3.2 Community cohesion and social support

This theme identifies participant perceptions of the impact of specific social determinants on their communities and the types of social support needs requested. Further, participants emphasized the importance of incorporating current and historical local context and values when developing future strategies to address local substance use and overdose.

Participants identified consistent differences in the quality of housing and education by race. For example, participants highlighted differences in the quality of resources as well as the lack of real improvement, particularly in public education.

“*Education is more powerful in the whiter schools than in the black. C'mon now, it's segregation. Who got the best jobs and who don't? You got kids in the eighth grade four years behind in reading…”* and public housing “*You gotta think. I grew up in these projects, right? I'm 47 years old. The aesthetics of these places haven't changed since I was a child… You want us to take pride in our community, pride in ourselves, but you haven't given us nothing to take pride in. We don't even have grass no more, right?”*.

Twelve participants also shared that the current set of solutions to address pressing issues, particularly around housing, have not necessarily resulted in positive, neighborhood-level changes to their community. Instead, they experienced such decisions as producing additional stressors.

“*Because when I come here and I see all of these buildings… these people can't afford this. What are they doing? They can't afford this. I mean, it's hard enough. Everything has been gentrified.”*.

Consequently, participants believed that resources for those at greatest need were being diverted away from the community.

Participants believed three classes of supports would improve community wellbeing. First, they indicated a need to address crime, although agreement on the resources needed to do so was mixed. Five individuals indicated that they thought the increase in crime reduced the number of neighborhood-facing events and activities, which had previously benefited the community. In turn, participants thought fewer positive options made it easier for community members to engage in negative actions.

“*Gang culture filled the void that was left when they took sports and all that stuff. You know, the neighborhoods, you know, it was replaced with something and that something was gang culture*.”

However, removing crime completely was considered difficult because participants acknowledged its role in the community economy:

“*So let me let me say devil's right. That hidden economy is feeding some families. Yes, that hidden economy paying rent. It's no way to counterbalance that, right? Because if you take it away and it's gone and… You'll kill off things. That will happen. The Great Depression*.”.

Second, 14 participants discussed a need for resources to build community wellbeing while acknowledging the culture shift from what they described as a “village” of shared social norms to a culture focused on individualism:

“…*so I didn't grow up in the city of Richmond or any projects, but we did grow up with my grandma was right here. My mom was right here and uncle was—… the village raised us. My parents worked so the village raised us. So guess what? If I didn't make it home before the lights changed? Oh my, my grandma see me outside. My grandma gonna tap my butt because I know better, right? And so, I think we also took back that it takes the village to raise them because now I can't correct the child after cussing me out.”*

Third, despite the perceived loss of a “village,” 12 participants detailed the important role of positive family and recovery communities in their or their loved one's recovery success. They discussed how these individuals and groups accepted their history of substance use, saw them as a person, and provided continued support throughout their recovery journey:

“*Your village is who you create. [Verbal participant agreement]. You have to surround yourself with your support system and it's really genuine. We're so into, we wanna mind our own business. That's what's driving it, because we're not looking out for the next person. You get what I'm saying? But when you start to come together, ‘No, I know you. What's going on? … Let's get you some help, I'll get you– I know this person. I'll connect you.' When it becomes more of a community involvement, I think you would see a lot of that stuff decrease*.”

In response to evolving social norms around parenting, substance use, and loss of community, 20 participants discussed the parental and familial effort needed to directly educate their children and one another across a breadth of topics. In response, participants discussed types of educational supports needed to address their gaps in knowledge, including: (1) parenting strategies, (2) resources to support their own education around substance use, and (3) strategies to support their loved ones' mental health through the recovery journey.

“*My nephew just died from overdose… it really tore my family … not apart like it just hurt them a little more… as in like we didn't do enough for him. That really impacted them really hard*. *We knew he had a problem. He was going to meetings, but he wasn't on anything to help him. So, he was kind of doing it of free will—just got out of jail, was brought out to the program…So we didn't know—his last time he had called his family when he felt like using and this time he didn't*.”.

## 4 Discussion

This study represents the one of the first to utilize community partnership and CBPR principles to compile Richmond resident perspectives around substance use. Further, this is one of only a few local-level qualitative examinations of substance use in a US city following the COVID-19 pandemic (14, 15, 21). While themes mirror previous findings, the persistence of these patterns reveal the ongoing critical gaps between academic knowledge and local interventions. By lifting local voices and lived experiences, this study provides insights and recommendations to drive local action and also more widely reinforces the importance of translating academic knowledge into community-driven strategies that build resilience and lasting change.

### 4.1 Supports for individuals using substances

#### 4.1.1 Corrections-related supports

Across conversations, participants identified the need for additional support for individuals in corrections facilities, specifically prevention-oriented approaches to addressing substance use. As several participants pointed out, local criminal justice settings provide legally mandated treatments (e.g., hospital-induced detox, temporary withdrawal management, and group counseling) (22); additional legislation may be needed to expand support. Inclusion of an array of approaches within legally mandated treatments and support to navigate to the best-fitting modality would also allow for a more person-centered approach to corrections supports. As a start, expanding access to medications for substance use disorder in both short-stay jails and long-term prisons can significantly improve recovery success following release (23). Enhancing mental and behavioral health services that promote healthy coping practices, as requested by participants across conversations, can also address underlying modifiable factors associated with substance use in prisons (i.e., co-occurring psychiatric conditions, insomnia, stimulus deprivation, and boredom) (24).

#### 4.1.2 Clinical care supports

Many participants identified limited oversight in reducing opioid over prescription as a factor contributing to substance use in clinical care settings. A number of states reported reductions in opioid prescription and misuse following the implementation of Joint Commission stewardship guidelines and prescription monitoring programs (PMPs) (25, 26). However, programs that broadly restrict opioid prescription without accounting for nuances in patient experience and clinician judgement can increase patients' risk of unmanaged pain and withdrawal (25). Use of data from PMPs to balance prescription practices may be most effective when paired with: (1) culturally and individually tailored treatment planning, (2) multimodal pain management strategies, and (3) collaborative care team involvement (27–29).

Several participants also called for local providers to incorporate alternative, non-pharmacological care approaches into existing substance use disorder treatment regimens through collaboration with other programs that provide a broader range of support (e.g., integrative medicine clinics, community centers, and creative arts therapy). Many participants identified specific activities (e.g., art, music, journaling, exercise) or engagement with peer groups as a means of re-directing time and energy that may have previously been focused on engaging in substance use. Although a growing body of literature supports these approaches' effectiveness in managing chronic pain and mental health (30–33), limited evidence currently exists to demonstrate these approaches' effectiveness in reducing substance use directly (34).

#### 4.1.3 Treatment and recovery organizations

The continued presence of unlicensed individuals targeting and selling substances in or around treatment and rehabilitation facilities was identified as a concern. Consequently, participants suggested the development of a public-facing facility and peer-review platform to readily access information on the internet as rated by client and provider feedback across multiple areas, including access to substances. While an “informed consumer” approach to increase facility accountability may be challenging to implement in practice, similar applications have been developed in the review of healthcare.

### 4.2 Community interventions for social cohesion and resource connection

#### 4.2.1 Neighborhood connection and communication

Participants articulated several actions to support neighborhood-level connection and communication that have been demonstrated to improve social cohesion (35), reduce violent crime (36), support unhoused populations (37), and improve health outcomes (38). Participant-identified actions included building on or expanding strategies that are being implemented to varying degrees in Richmond, including: culturally-informed health and wellness fairs (39, 40); local sports leagues (41); spaces for topical discussion and collective learning (42); free or low-cost shared meal opportunities (43, 44); offering a variety of child-centered activities (45); and balancing policing strategies to positively support people with varying levels of severity across substance use disorders while maintaining community safety (46). Residents also supported (1) long-term initiatives that apply “village” principles to offer education and guidance to parents, as well as (2) mentoring and activity programs to youth (e.g., Richmond Police Athletic League) (47, 48). These approaches have significantly improved youth and adolescent academic achievement and physical health (49) as well as self-reported wellbeing (47).

#### 4.2.2 Linkage to care

Participants and community service providers identified ability to pay, insurance eligibility, and Medicaid prior authorization practices as key barriers to service initiation. Offering rapid access (within hours or days of deciding to reduce or abstain from substance use) to services was identified as a need. In the absence of such support, many participants reported that they or those in their networks resumed substance use. The use of “bridge clinics” may help to mitigate that gap. Bridge clinics fill gaps in comprehensive care access for individuals experiencing substance use crisis or at critical points in care transition (e.g., reentry after incarceration, homelessness, or hospital discharge) (50). While one large hospital emergency department in Richmond offers same-day buprenorphine with short-turnaround follow-up with their addiction medicine clinic, broader bridge clinic implementation may help address the need (51, 52). Participants also requested “one-stop shops” with representatives from various governmental agencies and community organizations to support long-term resource connection (19, 53). While one such facility serves Richmond's East End (54), additional hubs across the city could increase access. Participants also noted that while city bus fare is currently free (55), the range in location and time-of-day offerings to areas with treatment facilities is limited. When paired with efforts to physically centralize resources, overarching transportation supports like curb-to-curb demand response services (56–58) and mobile resource vans (59) might increase convenience without compromising affordability.

### 4.3 Personalized approaches for information-sharing, training, and education

Participants emphasized the importance of recognizing and addressing the unique circumstances and diverse narratives within each individual substance use and recovery process while also leveraging the strengths of social media to broadly share tailored information and individualized educational resources. They prioritized three topics: (1) individualized harm reduction and treatment, (2) engagement of support systems in substance use, recovery, and coping, and (3) more accessible information to balance negative external influences.

Participants emphasized the need for greater support of the family unit and support system engagement in substance use treatment. The use of adaptive coping methods (60) (i.e., strategies to manage stress, reduce anxiety, and learn general social skills and assertiveness) were most effective when paired with support system assistance through Significant-Other Involved Treatments (18). Further, interrupting generational cycles of substance use could be addressed through tailored resource support for children, parents, and support systems may promote successful family-wide coping strategies following an adverse life event (61). These results offer a call to treatment providers for assessment of the health of the client's support system, as well as connection to resources such as peer support groups to preserve healthy, reciprocal support networks (62). These strategies may offer additional opportunities to support the networks surrounding a person engaged in substance use or recovery.

There was support for the use of consistent messaging and high-quality content through social media. Future strategies should consider collaboration with residents and the larger community to develop such content. For example, youth engaged as ambassadors to develop and disseminate prevention messages within their own social media networks and through in-school activities demonstrated reduced levels of eight areas of substance use, beliefs, and intentions (63). This success may extend to adults and may offer a unique opportunity for future messaging development.

### 4.4 Limitations

Study findings should be interpreted in light of the following limitations. First, participants were selected via convenience sampling. These results may not be representative of all experiences among people engaged in substance use throughout Richmond, Virginia. Second, it is not always possible to distinguish participants sharing personal substance use experience from participants speaking to the experiences of others due to study privacy protections. Thus, many of the conversations discussed drivers of substance use in general and not specifically the outcome of overdose. Third, the number of references coded for each main idea expressed in conversations was manually tallied and may differ based on individual interpretation, introducing the potential for researcher bias. Fourth, the discussion of specific topics was rotated across focus groups to ensure a wide range of perspectives. The themes identified may therefore reflect underestimates of the degree to which identified concerns affected participants. Additional self-report survey data may be warranted to assess population-level endorsement of concerns and to determine the likelihood of engagement for identified resource area needs. Nevertheless, many of the issues and resource needs identified here were also reported in Virginia, through treatment service utilization data and interviews with organizational stakeholders (64), and in other locales (64–66). Consequently, many of the results in this study are consistent with previously reported results.

### 4.5 Strengths

The core research team members participated in a survey and group reflection on project development and execution at the end of the project to identify strengths and lessons learned from the process. Seven of nine team members (77.8%) completed the survey, and six participants (66.7%) engaged in the group discussion. Project strengths included: (1) team member passion and willingness to support the community, (2) personal growth and knowledge that can be applied to future work or transferred to other types of work, and (3) project completion despite the newness of team members and changes in team dynamics. The team identified the following lessons learned: (1) a need for more detailed orientation at the beginning of the project; (2) having access to specific resources and training (e.g., CBPR principles, the research process, facilitation, public speaking, and communicating with data) throughout the life of the project, rather than at the project start; and (3) providing time and space for team members to develop effective working relationships through trust, clear communication, and respect. Further, a central aim of CBPR is to translate study knowledge into actionable and sustainable solutions. As a result, VCU, community partners, and RHHD have been incorporating study findings into their work (Table 6).

**Table 6:** Summary of projects implementing study results.

**Study theme**	**Project type**	**Description**
• Knowledge and information-sharing around substance use • Community- and institutional-level access to substances • Consistent wrap-around resource support	Community and local government stakeholder presentations	Results and process were shared within RHHD departments and Richmond-area stakeholders external to the health department between 2024 and 2025. Study findings are informing Richmond Overdose Task Force (ROTF) strategic planning. Initiatives such as harm reduction vending machines and youth substance use prevention education have been funded as a result^*^.
• Knowledge and information-sharing around substance use • Community- and institutional-level access to substances	Health department presentations	Results and process were shared across Virginia Department of Health departments and local health departments between 2024 and 2025 to encourage process implementation throughout Virginia.
Knowledge and information-sharing around substance use	National and state conferences	Results and process were shared in presentations at two conferences (National Association of City and County Health Organizations 360 the Virginia Epidemiology Seminar) to aid other local health departments conduct similar processes.
Consistent wrap-around resource support	Resource guide	A partnership between RHHD and the City of Richmond have developed a substance use services resource guide of community partner organizations, eligibility status, and other criteria to aid in navigation of these services (67).
Knowledge and information-sharing around substance use	Community education	The VCU team shared focus group at 10 community events from April–October 2025 using a Party with Data approach (68).
• Knowledge and information-sharing around substance use • Community cohesion and social support • Community- and institutional-level access to substances	Community education	Two community-wide substance use knowledge and education workshops were developed on (1) Myths and Facts about substance use, (2) Self-care and care for loved ones experiencing substance use, and (3) Resource connections to substance use specific and general community resources in collaboration with the focus group results as well as requests from non-profit stakeholders with the Peter Paul Community Action Network (CAN, https://www.peterpaulrva.org/community). Workshops reached all age groups in response to focus group feedback.
Knowledge and information-sharing around substance use	Community education	Focus group feedback requesting education and the need to develop communications skills around substance use guided the VCU team and RHHD to pilot on an after-school (8–12th grade) education program in December 2025 that provided substance use education paired with arts-based reflection using photo-voice techniques (69). The RHHD team uses educational lessons developed by the Virginia Foundation for Healthy Youth^*^.

^*^The Richmond Overdose Taskforce initiatives have been funded by the Virginia Opioid Abatement Authority.

## 5 Conclusion

Multiple evidence-based recommendations were identified to reduce opioid overdoses and increase holistic resource connection in Richmond, VA. The results underscore the gap between knowledge and action. Strategic investment in system-, community-, and person-centered supports, alongside interpersonal peer and familial support networks, encompass the multi-dimensional supports needed to prevent adverse outcomes associated with substance use. Beyond guiding local health district priorities, the above recommendations can inform future locality decision-making and program planning to build resilient communities.

## Data Availability

The datasets presented in this article are not readily available because data sharing agreement is needed with Virginia Department of Health to access the qualitative data. Requests to access the datasets should be directed to Melissa Viray, melissa.viray@vdh.virginia.gov.
