# Supplementary material for: A community conversation process to establish resident and service provider perspectives on needs related to use and treatment of opioids and substances
Source: Front Public Health. 2026 Jan 27;13:1678130. doi: 10.3389/fpubh.2025.1678130 (PMC12886460; doi:10.3389/fpubh.2025.1678130)
Supplement: Supplementary file 1 [file Data_Sheet_1.zip › Appendix B, Fig. B.4 (Study Consent Form).pdf]

Community Conversations: Overdoses in Richmond

Why do you think more people are experiencing overdoses in Richmond? What should we do about it?

What are we doing?

The Richmond and Henrico Health Districts — in partnership with FREE (Finding Redemption through Enlightening and Education), A Better Day than Yesterday, and Nolef Turns — want to gather input on what the community feels is driving overdoses in Richmond and what we should be doing about it. RHHD will then use the information to shape programs that may reduce overdoses.

Why now?

The number of people overdosing has more than doubled in Richmond since 2017. In fact, the percentage of Richmonders who overdose is higher than it is across the state overall. We have also seen an increased number of overdoses in individuals ages 20–34 in our region.

Who can participate?

Anyone 18 years or older who lives in the Richmond area can participate. We will not ask you to provide an address.

What will happen if I decide to participate?

You'll participate in an educational session, and then join an in-person conversation about substance use in our area with other members of the community. Individuals who participate will have the opportunity to share their own experiences and thoughts about substance use, overdose, and what supports the community needs most. Only the audio from the conversation will be recorded.

Food will also be provided at the session.

What should I know before participating?

Some questions we talk about in the community conversations are on difficult topics. Should you need them, study team members can refer you to resources to receive help. To keep everyone safe and follow best practices, if you choose to share about harming children, yourself, or others, we will provide additional resources and report information shared to an outside source.

What we **WILL** report	Examples of what we will **NOT** report
**Harm to children** will be reported to the Department of Social Services.<br><br>**Harm to self or others with intent** will be taken seriously and referred to appropriate resources.	- Current drug use<br>- Past drug use<br>- Violations of parole<br>- Gang Affiliation

How will you keep my information safe?

During the conversation, we will try to keep the use of names to a minimum. All identifying and personal information—like first names, last names, and nicknames—will be removed from any materials, including the recordings of the conversations. While we will keep a transcript of the conversations, the recording itself will be deleted. Our findings, including direct quotes, may then be shared with the community, other organizations, and partners.

If you choose to participate, please help us by respecting the privacy of other community members. Do not reveal participant identities or any personal stories and experiences discussed in the community conversation.

What will happen if I decide NOT to participate?

At any point you can leave the community conversation or choose not to answer a question. If there are thoughts you want to share, but not out loud, please fill out a card and put it in the Listening Box.

Who can I talk to if I need help or have questions, comments, concerns, or complaints?

Any questions, comments, concerns, or complaints about the project can be directed to:

- Jenn Shepheard, MSc, Population Health Epidemiologist, Sr at RHHD, jennifer.shepheard@vdh.virginia.gov
- Dr. Melissa Viray, Deputy Director at RHHD, melissa.viray@vdh.virginia.gov

Please print your name, date, and sign the form stating that you have read and understand the information provided above. If you have any questions please ask the event staff. Then please share your thoughts and experiences in the following conversation. If there are thoughts you want to share but not out loud, please fill out a card and drop it in the box.

Name:

Date:

Signature:
