# Supplementary material for: A community conversation process to establish resident and service provider perspectives on needs related to use and treatment of opioids and substances
Source: Front Public Health. 2026 Jan 27;13:1678130. doi: 10.3389/fpubh.2025.1678130 (PMC12886460; doi:10.3389/fpubh.2025.1678130)
Supplement: Supplementary file 1 [file Data_Sheet_1.zip › Appendix B, Fig. B.1 (Resource Connection Form).pdf]

RICHMOND CITY
HEALTH DISTRICT

Resource Centers

Working together for healthy people in healthy communities

Could you or your family benefit from access to the following services?

Please check all that apply

Medical Support Resources:

- ☐ Family Planning
- ☐ Eye Care
- ☐ Immunization Services
- ☐ Healthcare Insurance
- ☐ General Medical Care/Primary Care Physician
- ☐ Free and Confidential HIV/ STD testing

Other Resources:

- ☐ Pregnancy Resources
- ☐ Resources with the Department of Social Services
- ☐ Information about Employment
- ☐ Mental Health Support Services
- ☐ Dental Services
- ☐ Information about Education
- ☐ Housing Resources
- ☐ Rental Assistance
- ☐ Electric Assistance
- ☐ Food Resources
- ☐ Parenting / Child Care Resources
- ☐ Nicotine Replacement Therapy (NRT)
- ☐ Other ____________________

Today's Date: __/__/___ **What would you identify to be your Top 1 need?**

#1 need: ______________________________ Contact #: __________

Client Name: ______________________ D.O.B. __/__/___ Age: _____

Email: ________________ Address: ______________________

Do you have insurance? Y N If yes, what kind? Private/Medicaid/Medicare/Medicaid MCO

Do you have a Primary Care Provider (PCP)? Y N If yes, Dr. ______________

Have you seen your PCP in the last 6 months to 1 year? Y N If no, let's schedule an appt.

Where is your doctor's office? VCUHS Crossover CAHN Daily Planet Care-A-Van Bon Secours Health Brigade

Staff assigned: __________ Entered in CHWCC?: Yes
