# Supplementary material for: A community conversation process to establish resident and service provider perspectives on needs related to use and treatment of opioids and substances
Source: Front Public Health. 2026 Jan 27;13:1678130. doi: 10.3389/fpubh.2025.1678130 (PMC12886460; doi:10.3389/fpubh.2025.1678130)
Supplement: Supplementary file 1 [file Data_Sheet_1.zip › Appendix B, Fig. B.3 (Community Resource Brochure).pdf]

Accessible Substance Use Treatment Programs

Atlantic Outreach Group (AOG)

2421 Westwood Ave, Suite A
(804) 308-2648
https://www.aog4u.org/

AOG strives to empower vulnerable communities by connecting them to resources necessary for their success. Services include a food pantry (call to schedule a pick-up time and alert them of dietary restrictions/ allergies), a sober living program, and a full-service recovery program.

CARITAS

2220 Stockton Street
(804) 358-0964
https://www.caritasva.org/

CARITAS is home to an adult emergency shelter, a furniture bank (must be referred by one of their partner agencies), a long-term peer-driven residential recovery program for people recovering from substance use disorder, a recovery residential living community, and a workforce development program.

CleanSlate Center

1510 North 28th Street, Suite 101
(804) 521-0050
https://www.cleanslatecenters.com/

CleanSlate Center, a treatment center for those suffering from opioid or alcohol addiction, offers alcohol and drug rehabilitation through high-quality medication-assisted treatment in an outpatient setting. CleanSlate also offers a unique program for pregnant women or those who have recently given birth.

Job Resources

Virginia Career Works Capital Region

2121 Cedar Fork Rd Suite B.
(804) 652-3220
https://vcwcapital.com/

Virginia Career Works provides one-on-one assistance with resumes, cover letters, and more. There is also information and assistance for formerly incarcerated people returning to work, youth and veteran job seekers, career counseling, and more.

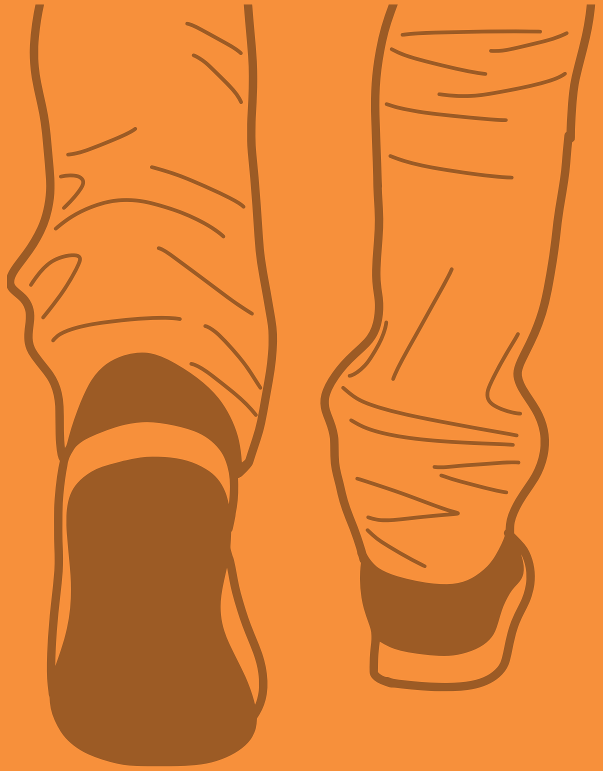

Taking Steps Forward

RHHD
RICHMOND & HENRICO HEALTH DISTRICTS

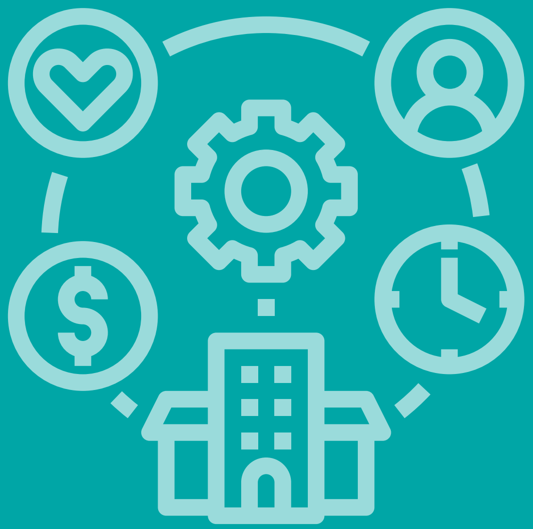

Community Resources *& Substance Use Support*

RHHD
RICHMOND & HENRICO HEALTH DISTRICTS

Social Support & Life Skills

National Counseling Group

25014 Monument Ave
(804) 497-4676
https://www.ncgcommunity.com/

National Counseling Group has services for mental, behavioral, and substance use and is located in several locations in Virginia. This include mental health skill-building, life coaching, intensive in-home services, community stabilization, and more.

RVA Light

2506B West Broad Street
(804) 447-7087
https://rvalight.com/

RVA Light is an organization that aims to connect people struggling with mental and physical illness, addiction, homelessness, unemployment, and food insecurity with resources. They host a variety of groups and classes designed to empower their neighbors to meet their personal goals, such as anger management classes, free haircuts, Bible study, mentorship through music for youth, and more.

Finding Redemption Thru Enlightenment & Education (F.R.E.E.)

Email: findingredemptionrva@gmail.com
(804) 263-6596
https://www.instagram.com/f.r.e.e.llc/

F.R.E.E. updates people in the Richmond community about a variety of events and resources, including job trainings, substance use treatment options, Narcan trainings, reentry services, and consumer loan opportunities. F.R.E.E. also provides adult peer support and workforce development for formerly incarcerated individuals.

A Better Day Than Yesterday

Email: info@abetterdaythanyesterday.org
(804) 299-1932
https://www.abetterdaythanyesterday.org/

A Better Day focuses on reunification and connection-building for families impacted by incarceration, divorce, and military deployment. Their programs help adults and children adjust to new lifestyles and family structures through communication workshops, goal-setting practice, bonding activities, and more.

Nolef Turns

2317 Westwood Ave. Ste. 209
(804) 918-6470
https://www.nolefturns.org/

Nolef Turns empowers and advocates for those who have been affected by crime, trauma, and incarceration. They offer pre-release, re-entry, and post-conviction services to prepare people for success at each step of the way. Some of these services include shelter, clothing, and food referrals, entrepreneurship, money management, and technology assistance. Nolef Turns also provides family support services like college/trade program prep, homework help, and 1-on-1 mentoring to children of incarcerated parents.

Financial Literacy & Housing

REAL LIFE

Email: info@reallifeprogram.org
(804) 406-4111
https://reallifeprogram.org/

REAL LIFE serves individuals who have been impacted by incarceration, homelessness, or substance use disorder by giving them an opportunity to overcome their personal and community barriers. They offer a program at no cost, which includes moving into their recovery transitional house. People will attend classes, including anger management, parenting, financial literacy, and more, for several months to get on the path toward a thriving life.

Richmond Redevelopment and Housing Authority (RRHA)

901 Chamberlayne Parkway
(804) 780-8700
https://www.rrha.com/

RRHA helps individuals and families transform their lives by providing real estate development, rental housing assistance, and property management of public housing communities for low- and moderate-income families, seniors, and people with disabilities throughout Richmond. They are also the largest public housing authority in Virginia and provide subsidized housing assistance.

Forward Foundation

3751A Westerre Parkway, Suite 103
(804) 840-8687
https://forwardfoundationva.org/

Forward Foundation assists working, single parents and their children in the Greater Richmond area who are in the midst of a financial crisis. Their program requires completion of educational workshops led by community partners. They also provide families with education resources, tools, and community support.

Housing Opportunities Made Equal (HOME) of Virginia

626 East Broad Street, Suite 400
(804) 354-0641
https://homeofva.org/

RHOME aims to ensure equal access to housing for all people. Services include fair housing, homeownership, foreclosure prevention, eviction prevention & diversion, and a Move to Opportunity program to help those with housing choice vouchers rent housing in neighborhoods of opportunity. Must apply for services.

Public & Private Sector Support

CommonHelp

(855) 242-8282 (Healthcare Coverage)
(855)522-5582 (To Apply for All Benefits)
https://www.commonhelp.virginia.gov/

CommonHelp is a resource for people in VA to check the status of their benefits, report changes to their status, and find out if they may be eligible to receive energy assistance (fuel, crisis, or cooling), food assistance (SNAP), financial aid (TANF or auxiliary grants), or health insurance. People can also search for the closest Department of Social Services near them.

Go To Work

Email: info@gotowork.app
(804) 548-4310
https://gotowork.app/

Go To Work is a prepaid, prescheduled, human services rideshare transportation program. Their services include providing rides to work, job skills training, and daycare, saving riders on average 50% the cost of Uber and Lyft. Must register for the program in order to schedule rides.
