# Supplementary material for: A community conversation process to establish resident and service provider perspectives on needs related to use and treatment of opioids and substances
Source: Front Public Health. 2026 Jan 27;13:1678130. doi: 10.3389/fpubh.2025.1678130 (PMC12886460; doi:10.3389/fpubh.2025.1678130)
Supplement: Supplementary file 1 [file Data_Sheet_1.zip › Appendix A, Table A.1 (Question Inventory).pdf]

Table A.1. Conversation Question Inventory

**Conversation Questions**	
Drivers of Overdose Increase	• Name all the drugs you know of.<br>• Which substances do you think are the easiest to get and why?<br>• How do you feel about opioid use in your community?<br>• How do you feel when you hear about an overdose in your neighborhood/area?<br>• How has substance use impacted you, your family, and your community?<br>• What challenges do you face after a loved one has overdosed?<br>• How does seeing or hearing about an increase in people who overdose in the community change the way you interact with your community and/or use drugs?<br>• How do you feel about the use of methadone and suboxone (MAT) for treatment?<br>• What have you seen that has increased substance use in your community?<br>• Why do you think Richmond has had more overdoses?<br>• Do you feel that certain age groups [or demographics] are more strongly affected by substance use in Richmond than others? If so, which ones?<br>• Why did you start using?<br>• Name all the reasons you do not use drugs.<br>• What prevented you from recovering, decreasing, or stopping use?<br>• Do you have anything you like to do that takes your mind off using?<br>• What do you do after work or school? Does that stop you from using?<br>• How does your method of relaxation change your substance use, if at all?<br>• What activities are you involved in that reduce your stress?
Resource Provision and Need	• What substance use resources (supports) are in your area?<br>• What programs, people, activities, or resources make you feel most supported?<br>• What programs, resources, community members, etc. prevented you from using or starting to use?<br>• What resources would you like to see? (What do you need help with?)<br>• What barriers can you think of that might prevent people from accessing treatment, even if they feel ready to go?<br>• In your opinion, how can we reach more people in Richmond about existing programs and resources?
**Dissemination Questions**	
Drivers of Overdose Increase	• In the last three months, do you think more people used opioids, prescription opioids, or cocaine?<br>• Do you think that people's responses about [x topic] might differ based on whether they have personal experience with substance use?<br>• Do you see any support factors that you think could also be deterrents (i.e., strong enough to turn people away from substance use on their own?)<br>• We have MAT (medication-assisted treatment) down as a support. Do you think this is right? How does your community perceive MAT?
Resource Provision and Need	• If you could only choose one, which substance use resource area would you prioritize in Richmond: Prevention, Recovery Support, Treatment, or Harm Reduction?<br>• If you could only choose one, which general resource area would you prioritize in Richmond: Job Resources, Housing Resources, Social Support and Life Skills, Personal Finance Education, or Better Support from Government or Businesses.<br>• What do you think we could do to reach more people with personal experience using substances and connect them with existing resources and programs?
