# Supplementary material for: A community conversation process to establish resident and service provider perspectives on needs related to use and treatment of opioids and substances
Source: Front Public Health. 2026 Jan 27;13:1678130. doi: 10.3389/fpubh.2025.1678130 (PMC12886460; doi:10.3389/fpubh.2025.1678130)
Supplement: Supplementary file 1 [file Data_Sheet_1.zip › Appendix D, Tables D.1-2 (Summary Results).pdf]

Table D.1. Summary of Results Related to Drivers Increasing Substance Use Overdose

Theme	Subtheme	Code
Diversity in Substance Use Narratives	Drug Characteristics	Defining a Drug
	Substance Use Patterns and Practices	Drugs of Choice, Methods of Use, and Affected Populations
	Demographic Influences	Age Group and Generational Affiliation
		Racial and Ethnic Identity
		Gender Roles and Expectations
	Individual Perceptions and Discourse Around Use	Conceptualizing Use
		Conceptualizing Recovery
		Harm Reduction Attitudes
Community- and Institutional-level Access to Substances	Social Atmosphere Around Use	Characterizing the Local Climate
		Cultural Norms, Hierarchies, Stereotypes
	Legislative and Political Dimensions	Drug Manufacturing and Market Regulation
	Drug Characteristics	Access and Availability
		Formula and Potency
		Variety
	Income and Financial Stability	Compensation and Costs
	Community Composition	Social Influence and Group Belonging
	Criminal Justice System	Prison Environment
	Clinical Healthcare and Treatment	Prescription Practices
Coping with Impactful Life Events and Experiences	Impactful Life Events and Experiences	Sociocultural Contexts
		Physical Living Conditions
		Facing Grief and Loss
		Enduring the COVID-19 Pandemic
	Substance Use Patterns and Practices	Situational Influences on Use Behaviors
	Coping Strategies	Self-Medicating
		Engaging in Hobby Activities or Past Times
		Practicing Mindfulness and Internal Reflection
		Managing Social Connectivity
	Psychological Experiences	Mental Health and Cognition
		Emotional Health and Resilience
		Self-Concept and Awareness
		Sense of Safety
	Physiological Experiences	Seeking Homeostasis
		Altering the Body and Mind
		Genetic Risk and Family History
	Physical Health	Co-occurring Health Conditions or Injuries
	Faith and Spiritual Health	Religious Involvement and Spirituality

Table D.2. Summary of Results Related to Community Resource Requests

Theme	Sub-Theme	Code
Knowledge and Information-Sharing around Substance Use	Transmission of Substance Use Knowledge	Interpersonal Role Models
		Pop Culture, Music, and Media Representations
		Educational Programs and Public Advertising Campaigns
	Community Education and Awareness	Drug and Substance Use Knowledge
		Health Literacy and Health Behaviors
		Systems Navigation and Advocacy
		Formal Education and General Literacy
Community Cohesion and Social Support	Legislative and Political Dimensions	History of the Public Education System
	Built Environment	Urban Planning and Restructuring
	Community Composition	Family Dynamics and Involvement
		Neighborhood Relations and Community Cohesion
Consistent Wraparound Resource Support	Built Environment	Housing Access and Affordability
		Transportation Systems
	Employment	Workforce Entry Requirements and Pathways
		Job Aspects and Attributes
	Income and Financial Stability	Childcare Coverage
	Legislative and Political Dimensions	Approaches and Outcomes of the “War on Drugs”
		Governmental Roles and Responsibilities
		Health Insurance Access and Utilization Management
	Criminal Justice System	Interfacing with Law Enforcement
		Lasting Effects of Incarceration
	Clinical Healthcare and Treatment	Care Access and Admission Process
		Nature of Service Provision and Support
		Care Transition
	Local Resource Landscape	Community Resource and Service Provision
		Government-Sponsored Support System
		Resource Connection and Engagement
