# Supplementary material for: A community conversation process to establish resident and service provider perspectives on needs related to use and treatment of opioids and substances
Source: Front Public Health. 2026 Jan 27;13:1678130. doi: 10.3389/fpubh.2025.1678130 (PMC12886460; doi:10.3389/fpubh.2025.1678130)
Supplement: Supplementary file 1 [file Data_Sheet_1.zip › Appendix C, Table C.1 (Qualitative Codebook).pdf]

**Table C.1. Qualitative Codebook**

| Subtheme | Code | Definition | Decision Rule | Example | First Occurrence |
|---|---|---|---|---|---|
| Drug Characteristics | Defining a "Drug" | Factors that inform people's understanding of a substance as a "drug," including legality, prescription status or medicinal use, and moral evaluation | What makes a drug a "drug," as opposed to a medication, supplement, natural remedy, etc; do not code statements that reveal nothing about what the word "drug" itself connotes | "If it ain't prescribed to you and you ain't supposed to take it, then it's definitely a drug."; "Can I ask about the word, 'opiate,' is that the pill or a drug?" | 19-May |
| | Formula and Potency | The chemical constitution and strength of a drug; changes and variability in drug composition (i.e., "cutting" one substance with another) | Code any mention of what physically makes up a "drug" (i.e., substance) here | "You got fentanyl in weed, you got fentanyl in pills, you got fentanyl in everything. And when they hit you, they hit you. I'd be dead, you know?"; "[A dealer] will say, 'yeah, I got what you want,' and then serve [people] anything. Don't even have to be the drug." | 19-May |
| | Variety | The diversity in names used to refer to a given substance; changes and variability in the drug supply (i.e., the advent of new substances) | Code all mentions of drug variety (i.e., what's on the market, what street or prescription names are used to refer to different drugs), with the exception of "variety" in drug composition, which would fall under Formula and Potency | "In the country we call girl soft and heroin boy. I mean no - we call crack boy and soft girl, but up here [in Richmond] they call hard girl and boy heroin."; "'I found this thing as I was on TikTok; it's called gas station heroin now,' ...'the xylazine, that's what it is.'" | 19-May |
| | Access and Availability | Factors that influence the ease of obtaining drugs in the local community; various sources and settings | Code any mention of where local residents can go to get substances, including but not limited to community social ties, online media sites, and physical | "I know at least three corner stores that I could walk into and get an ounce of weed — a pound of weed — from the local bodega."; "They have [pop-ups] all around Richmond… Instagram: that's | 19-May |

| Subtheme | Code | Definition | Decision Rule | Example | First Occurrence |
|---|---|---|---|---|---|
| | | where drugs can be found | locations; also include intermediary influences on individuals' ability to obtain drugs (e.g., cost, transportation) | where you mostly find them at."; "So now we're talking about dispensaries right? … Most parents aren't choosing that option because they can't afford it." | |
| Substance Use Patterns and Practices | Drugs of Choice, Methods of Use, and Affected Populations | Perceptions around local use practices, including commonly used drugs and method of use (i.e., inhalation, ingestion, injection, etc.); patterns of perceived variation in use across local population groups | Code any participant observations or assumptions around local substance use statistics (who is using, what is being used, how are people using, etc.) | "Alcohol is becoming one of our big consumptions right now since COVID hit. Our folks may not be using but they are shoving them back."; "A lot of older people aren't into meth like the younger people are. I think it depends on the drug." | 19-May |
| | Situational Influences on Use Behaviors | Situational factors that influence people's habits, choices, and practices with regards to substance use; events or situations that turn people towards or away from use | Do not code life events or experiences here unless the participant clearly links said event or experience with a increase or decrease in substance use | "My husband overdosed. He passed away and then I started using… because I thought if I got the right stuff I could kill myself."; "Housing is causing some of our folks to actually use… because they don't have it." | 19-May |
| Demographic Influences | Age Group and Generational Affiliation | Mentions of age or generation with regards to substance use, prevention, and interpersonal or community dynamics | Code any mention of numeric age, age group, or generational comparisons | "We're feeling like it's the younger ages [more affected by substance use in Richmond], 16 to 25 is what we're seeing."; "…start teaching our youth early… so you can stop the cycle. Because they grow up to be what they see…" | 19-May |
| | Racial and Ethnic Identity | Mentions of race or ethnic identity with regards to substance | Code any mention of racial group, ethnic group, or race/ethnicity as a factor in | "Look who's in the prisons: minority men! They want to see us destroy ourselves and each other."; "…it's | 19-May |

| Subtheme | Code | Definition | Decision Rule | Example | First Occurrence |
|---|---|---|---|---|---|
| | | use, prevention, and interpersonal or community dynamics | larger historic or societal patterns | not just the African American communities… it's happening in Caucasian households, Hispanic households. We just don't know because it's… not talked about." | |
| | Gender Roles and Expectations | Mentions of gender identity or gender-based assumptions with regards to substance use, prevention, and interpersonal or community dynamics | Code any mention of gender, sexual orientation, or biological sex with regards to an individual's character, motivations, or societal role | "Some women [don't enter recovery] because they have low self esteem… feeling unloved makes them do [drugs]…"; "Mental health is a key contributor [to drug use]. People aren't as informed about mental health and the effects of it… especially men. They feel embarrassed." | 13-Sep |
| Social Atmosphere Around Use | Characterizing the Local Climate | Community attitudes towards – and perceived level of acceptance around – substance use; the presence or absence of stigma; openness and frequency of substance use-related conversations | Descriptions of the broader, societal conversation around and view towards substance use | "There seems to be a message… an idea that it's not particularly accepted or okay to use [substances]…"; "I think the community should start doing things, not just on special nights, to talk about opioids. I think we should make it a regular conversation..." | 19-May |
| | Cultural Norms, Hierarchies, and Stereotypes | Common narratives that frame or exemplify community attitudes towards use; social distinctions and assumptions made with regards to people who use substances | Specific stories or examples that play into larger societal views on substance use; statements may be double coded to the Conceptualizing Use category if they reveal opinions held by participants, but not if they reflect views heard or espoused from outside community members | "I've been in some substance abuse spaces and heard people who battle with crack addiction argue with those who have cocaine addiction, and then they gang up on those who have opiate addiction... it's classism..."; "…Usually, drugs will only attack people who really have something to offer the world… some of the most intelligent people I ever met were drug addicts." | 19-May |

| Subtheme | Code | Definition | Decision Rule | Example | First Occurrence |
|---|---|---|---|---|---|
| Individual Perceptions and Discourse Around Use | Conceptualizing Use | Language used to describe, understand, and explain an individual's substance use; personal testimonies around initiating and sustaining use | Code any terminology participants use to describe substance use as a concept; include passages that give insight into the way people view use (i.e., why individuals use, what being in active use is like, etc.) | "It may be one of the most ungodly diseases on the face of the planet..."; "There's other barriers like finances, housing kids, all of that. Not saying it's not the opioids, I'm just thinking of other stuff that led to the issue." | 19-May |
| | Harm Reduction Attitudes | Attitudes towards harm reduction principles and practices (i.e., overdose prevention, medication- assisted treatment) | Code any discussion around the purpose, effectiveness, or accessibility of Narcan, drug testing strips, needle exchange, methadone, suboxone, etc. | "[MAT] makes you a functioning addict."; "It's a trade off with sudden death."; "Methadone will give you your life back."; "Harm reduction? I feel like we already have that." | 19-May |
| | Conceptualizing Recovery | Language used to describe an individual's recovery process; personal testimonies around recovery, including positive and negative influences | Code any terminology participants use to describe recovery from substance use as a concept; include passages that give insight into the way people view recovery (i.e., what prompts people to quit, what helps an individual succeed in maintaining recovery, etc.) | "You can pray, you can throw holy water on them, they can go to 50 treatment centers and it's not going to help until that person gets sick and tired. That's the bottom line."; "It's such a mental fight, a fight within yourself… some of us are gonna win, some of us are gonna lose. You just have to want it." | 19-May |
| | Outlook on the Future | Expressions of hope, hopelessness, or apathy towards the future of substance use and overdose prevention in Richmond | Code statements that reveal individuals' views on and attitudes towards the future, whether positive, negative, or neutral | "We just need to admit that… society has failed us, especially low-income communities."; "It just seems like all of it is a Catch 22… you don't qualify for the services or you do qualify for the services, but they can't help you… they won't help." | 2-Jun |

| Subtheme | Code | Definition | Decision Rule | Example | First Occurrence |
|---|---|---|---|---|---|
| Community Education and Awareness | Drug and Substance Use Knowledge | Individuals' knowledge and beliefs around substances, dosing, physical tolerance, overdose prevention strategies, and other experiences associated with use | What people know, don't know, or want to know more about; code anything related to substance use, drug characteristics, recovery, harm reduction, etc. | "A lot of times, you don't know what you're buying…"; "[Fentanyl] is just seeping into everything and people aren't realizing that they're using these even stronger forms of drugs, when they may think they're just recreationally using." | 19-May |
| | Health Literacy and Healthy Behaviors | Individuals' knowledge and beliefs around mental and emotional health, coping, and healthy lifestyle choices | What people know, don't know, or want to know more about; don't code coping activities themselves here unless accompanied by discussion of individuals' knowledge around them | "I feel like raising awareness and … having coping mechanisms for when life stressors happen, especially if you're somebody who feels alone..."; "…she did lose a child, so that might've been— she's grieving and she doesn't know how to grieve, but it's teaching her that..." | 2-Jun |
| | Systems Navigation and Advocacy | Individuals' knowledge and beliefs around finding, accessing, and utilizing existing services and resources | What people know, don't know, or want to know more about when discussing how to connect to, interface with, or continue resource support from the client's perspective | "I was looked at as if I didn't need help because I was well-spoken… I had to break it down and tell them what I was using, how often I was using, how I would put that over kids… my responsibility, my health, over everything… That's when they took me more seriously." | 19-May |
| | Formal Education and General Literacy | Individuals' knowledge and beliefs around the skills and competencies associated with traditional school environments | What that people learn, don't learn, or want to learn more about in school; if specifically discussing drug and substance use education in schools, code to Drug and Substance Use Knowledge instead | "So [resource and service providers] get trained, you go to school, you get all your credentials, you do all this stuff, but they don't train honesty, diligence, integrity… They don't train all that in school."; "There's so many [people in Richmond] that can't read." | 2-Jun |

| Subtheme | Code | Definition | Decision Rule | Example | First Occurrence |
|---|---|---|---|---|---|
| Transmission of Substance Use Knowledge | Interpersonal Role Models | Proximal storytelling and/or modeling of substance use knowledge, attitudes, and behaviors by peers, family members, neighbors, etc. | Informal, personal sources of substance use knowledge | "If you're telling me as a parent not to do [drugs], but at the same time you're smoking a cigarette or you're drinking…"; "It's the education [kids] get at home." | 2-Jun |
| | Pop Culture, Music, and Media Representations | Framing of substance use by celebrities, musical artists, news media, social media, and other online influences | Culturally-informed, public sources of substance use knowledge (formal or informal) | "All of them kids grow up with… all of them are easily influenced by this hip hop music right now that's telling them to go and get some Promethazine and all of this stuff." | 19-May |
| | Educational Programs and Public Advertising Campaigns | Structured education provided in school settings, corporate advertising campaigns, and public service announcements around substance use | Formal, public sources of substance use knowledge | "I mean, we had the D.A.R.E. program. I don't remember shit from that program except my officer's name." | 2-Jun |
| Local Resource Landscape | Resource Connection and Engagement | The effectiveness of resource communication methods and networks in reaching community members, fostering trust, and encouraging meaningful resource utilization | The "connection" piece of resource provision (i.e., spreading the word); do not code mentions of resource quality or service gaps themselves | "They give you a bus ticket and $25 until you come back. You have no resources, no family, no solutions to any questions you have… and you don't even know how to navigate the resources because they just give you a sheet, right? … It's bigger than just telling you where the resources are. | 19-May |
| | Community Resource and Service Provision | Community attitudes towards and experiences with existing community | Resource supports provided by community organizations and local service providers; code all | "[We need] a one-stop shop. Imagine if that building had everything in there… a rep from social services, somebody from the | 19-May |

| Subtheme | Code | Definition | Decision Rule | Example | First Occurrence |
|---|---|---|---|---|---|
| | | resource supports; individuals' assessment of broad community resource needs; explicit resource requests | participant resource requests here; government supports can be double coded here if they are part of a resource request and/or involve broader cooperation between community orgs and government | health department… someone from every facet."; "There's people running these programs that shouldn't be." | |
| | Government-Sponsored Support System | Community attitudes towards and experiences with government-provided benefits, assistance programs, resource supports, and funding opportunities | Resource supports provided explicitly by the government; content, quality, and accessibility of governmental supports | "You know what RRHA does? They do these swaps, right? So every couple years they're gonna uproot you and put you into a neighborhood that's totally foreign to you." | 19-May |
| Legislative and Political Dimensions | Governmental Roles and Responsibilities | Expectations for the role of local, state, or federal government officials with regards to substance use response and community interaction | Duties associated with the people working in government (e.g., shaping policies, writing legislation, allocating funding); do not code references to government-sponsored resources if there is no accompanying discussion around the roles, priorities, or actions of political leadership | "As a government, we got to take better care of our people."; "The system has well known what the issue is… how about you just follow through with all 10 recommendations. Don't pick and choose, 'I'mma do #1, #3, #7.'" | 13-Sep |
| | Approaches and Outcomes of the "War on Drugs" | Federal substance use policies and their impact on local level substance use patterns and response | Statements about how current criminal justice system and clinical substance use treatment practices were shaped; code any mentions of | "I've seen this. I've been in jail. A lot of people in jail ought to be in a treatment hospital. When are you going to try to treat that person? … but instead you give them life [in prison]." | 19-May |

| Subtheme | Code | Definition | Decision Rule | Example | First Occurrence |
|---|---|---|---|---|---|
| | | | substance use criminalization policies here | | |
| | History of the Public Education System | Discriminatory education policies and their impact on local public school education quality and student outcomes | Statements about how current education practices were shaped; do not code mentions of specific educational content or knowledge transmission methods (these would fall under Community Education and Awareness and Transmission of Substance Use Knowledge, respectively) | "Education is more powerful in the whiter schools than in the black. Come on now, it's segregation…" | 24-Oct |
| | Drug Manufacturing and Market Regulation | Governing bodies' oversight of the production, distribution, and consumption of drugs; corporate influences on legal and illegal drug markets | Discussion around major players in drug sales and trading (i.e., Big Pharma, marijuana dispensaries, cartels) | "We have a whole hidden economy… it's plaguing our community."; "That hidden economy is feeding some families. That hidden economy is paying rent." | 13-Sep |
| | Health Insurance Access and Utilization Management | Government insurance programs' implementation of prior authorization practices with regards to clinical service and substance use treatment | References to obtaining health insurance coverage and receiving approval for requested care services | I have submitted authorizations to MCOs and I had one sent back to me that I can provide 4 days of treatment, 3 hours a day, for 30 days. I said, '30 days?' They take 30 days to get on it… to get hooked." | 17-Nov |
| Clinical Healthcare and Treatment | Care Access and Admission Process | Experiences navigating clinical service and treatment; perceptions around | Interfacing with healthcare services prior to treatment enrollment or service provision; do not code statements about | "My brother was a heroin user and I took him to [treatment] quite a few times… I'll get him in the car, we'll drive all the way there, he'll go through the process, and if his | 2-Jun |

| Subtheme | Code | Definition | Decision Rule | Example | First Occurrence |
|---|---|---|---|---|---|
| | | eligibility and entrance requirements | community resource access, only access to clinical care; code statements about insurance access and approval here as long as they directly inform clinical service access | blood pressure is too high, they won't accept him." | |
| | Nature of Service Provision and Support | Attitudes towards treatment program effectiveness and facility environment; level of satisfaction with quantity and quality of care provided | Clients' experiences with healthcare facility environments, treatments, or services while receiving care; do not code statements about community resource provision, only clinical care services | "I know [someone who] went and got help several times through treatment, you know having a one-on-one counselor and everything, but he just felt like they wasn't genuine about it… just more of trying to get their money, basically, off his own pain." | 19-May |
| | Care Transition | Perspectives on the continuity of care and "wrap-around' support through periods of transition between clinical providers, services, settings, and resources (i.e., the external referral process) | Clients' experiences with healthcare system elements after receiving care or in between treatments; code here if clinical care is received on at least one side of the referral (e.g., social work referral to physician; social work referral to community resource, etc.) | "This is why I only work with people I trust as far as referrals go... You have people and you'll refer them and they'll just process them through the system… just do that initial intake, that crisis, and they just dump them off… and they're still billing!" | 13-Sep |
| | Prescription Practices | Views on pharmacological pain management, alternative medicine, and physicians' role in stewarding patient medication access | Medication or drug access through legal channels, most commonly physicians in clinics or hospitals | "Hospitals get people on, they put you on stuff that you could overdose… that might actually relieve the pain but you would still be okay with maybe a higher dose of Ibuprofen." | 7-Jul |

| Subtheme | Code | Definition | Decision Rule | Example | First Occurrence |
| --- | --- | --- | --- | --- | --- |
| Criminal Justice System | Interfacing with Law Enforcement | Attitudes towards and experiences interacting with police, parole officers, and other law enforcement officials | Individuals' experiences with law enforcement prior to, following, or entirely separate from incarceration (i.e., interactions that occur outside of prison) | "My mom was killed and her dad was killed. I won't even discuss how it happened… but I know it wasn't no authentic safety to go to because I tried. I made a beeline to RPD. Big zero. I got so low about no authentic safety..." | 13-Sep |
| | Prison Environment | Descriptions of life inside prison, including facility management and administration, economic conditions, and substance use-related experiences | Individuals' experiences with substance use and/or activities of day-to-day living during incarceration (i.e., inside prison) | "People need to be on suicide watch while detoxing in jail…"; "I got a jail call not too long ago from a friend… and he was telling me how somebody OD'd in the prison…" | 2-Jun |
| | Lasting Effects of Incarceration | Perspectives on the continuity of resource and service support through periods of re-entry after incarceration; discussion around pathways to continued criminal justice system involvement | Individuals' experiences with substance use and/or activities of day-to-day living following incarceration (i.e., after being released from prison) | "The individual I'm working with now, he was released in 2009. He's had some relapses, extensive history of trauma, but he just got permanent housing in the past two years. He's been out since 2009…" | 19-May |
| Employment | Workforce Entry Requirements and Pathways | Experiences seeking employment and navigating the job application process; perceptions around employment eligibility | References to seeking employment | "…if you want me to go get a job when I didn't take a shower in like four days… you know, I'm just keeping it a hundred." | 19-May |
| | Job Aspects and Attributes | Discussion around the opportunities and experiences provided through a job | References to being employed or having employment | "I chose 'Job Opportunity' because I feel like to build self-esteem… where there's something you work for that you value…"; "You need the | 7-Jul |

| Subtheme | Code | Definition | Decision Rule | Example | First Occurrence |
|---|---|---|---|---|---|
| | | | | routine job, you know, the routine of social interactions with people outside of the recovery house." | |
| Income and Financial Stability | Compensation and Costs | The financial factors and considerations surrounding substance use and recovery-related experiences | Include all mentions of cost (cost of street drugs, cost of tx programs, etc.), as well as behaviors driven by the desire for income | "Whoever's selling just trying to make the most money."; "Unless you spending $35 or more a pill, you ain't getting nothing on the street." | 19-May |
| | Childcare Coverage | The financial circumstances around and implications of childcare access | Include all mentions of childcare-related responsibility and opportunity cost of not having affordable childcare, even if financial cost of care is not directly discussed | "When you get to [a substance use treatment] appointment… they're going, 'Okay, we can help you. Go to wherever, do this, do that,' well who's gonna take care of my kid when I gotta do this?" | 2-Jun |
| Built Environment | Housing Access and Affordability | Mentions of affordable housing availability, homelessness, shelter space, and determinants of housing eligibility | Include discussion around the cost of rent, availability of existing infrastructure, etc.; distinct from "Sociocultural Contexts," which may include more personal or experiential descriptions of homelessness | "So I ride down [local avenue], I call for a studio apartment? $775. But this is the kicker, they have to prove they make three times that amount of rent."; "I think I get a voucher in 2028. Like what the hell? Until 2028 to find housing." | 19-May |
| | Urban Planning and Restructuring | Mentions of local population density, demographic shifts, and geographic changes or disparities in resource provision | Code mentions of and discussion around gentrification here; do not code specific descriptions of the physical neighborhood environment (Physical Living Conditions) | "I'm not even from Richmond, but to be here 20 years I have seen a significant change in the city... When I first came down here in the early 2000s, for one, it wasn't this dense." ; "So now you got transplants, people just not from around here occupying the space…" | 19-May |

| Subtheme | Code | Definition | Decision Rule | Example | First Occurrence |
|---|---|---|---|---|---|
| | Transportation Systems | Discussion around the presence and use of public or private transit; effectiveness of local geographic navigation methods | Include discussion around the implications of not having adequate or accessible transportation options | "…you normally got to go and show up at another place that may be 5, 10, 15, 20 miles on the other side of town, right... or there is no job. You got to go to Timbuktu." | 19-May |
| Impactful Life Events and Experiences | Sociocultural Contexts | Mentions of the social, cultural, and relational health of an individual's current or past environment (i.e., presence or absence of trauma, abuse, substance use, etc. as an adult or in childhood) | The general level of health or harm present in the interpersonal environment; double code between here and Community Composition as needed to specify/further characterize the nature of the relationships at play (i.e., if mention of abuse by family member, code here and to Family Dynamics and Involvement) | "You've seen it growing up your whole life… people laying down at the store, all the way to the ground, pants about to fall off..."; "There are a lot of people hurting inside them [homeless] shelters… a lot of things happen inside these shelters…"; "I was sex trafficked by people I thought really cared about me." | 19-May |
| | Physical Living Conditions | Mentions of physical environment's cleanliness, appearance, and overall favorability for individual health and wellbeing (e.g., presence or absence of physical gathering spaces, quality of spaces) | Do not include mentions of larger level population shifts and changes (Urban Planning and Restructuring), only tangible elements of one's physical surroundings | "I grew up in these projects, right? …The aesthetics of these places haven't changed since I was a child."; "I asked a lady… and she says, 'All I ever wanted to do was paint… I would just like to see some paint on the wall, some grass in the front so my grandkids can at least come and sit...'"; "[People] are not animals. They should not live like this." | 13-Sep |
| | Facing Grief and Loss | Experiences around loss or separation from a loved one, neighbor, or other personal relationship due to death, | Do not include experiences that causes someone to grieve if not defined by the physical loss of a person (i.e., grief associated with a domestic violence situation | "Me and my husband were on these streets together, and unfortunately two years ago he passed away from a drug overdose. And I wish I could say that's when I got clean… it | 19-May |

| Subtheme | Code | Definition | Decision Rule | Example | First Occurrence |
|---|---|---|---|---|---|
| | | overdose, incarceration, physical absence, etc. | would fall under Sociocultural Contexts) | wasn't. That's when I lost my mind completely." | |
| | Enduring the COVID-19 Pandemic | Unique experiences, social conditions, and substance use outcomes associated with quarantine and isolation practices during the COVID-19 pandemic | Include any mention of COVID-19 and accompanying description of how life looked during or after the pandemic | "So COVID hit. Now nobody can go anywhere… stay in your house and do nothing, be in your head. Then COVID ends. Now all those benefits that you were getting because you weren't working? I guess we're gonna take those away now." | 2-Jun |
| Coping Strategies | Self-Medicating | Coping through self-directed substance use; motivations and purposes for self-medicating (pain management, escapism, etc.) | Do not include discussion around over or under-prescription of medication (Prescription Practices) unless accompanied by mentions of self-directed use | "I think… we can't get professional medication, so we try to medicate ourselves and we do it by smoking weed, drinking, etc."; "I've been seeing, from a clinical standpoint as a nurse, a lot of self-medicating to try to deal with the feelings that they can't necessarily express, or feel like they don't have the resources to deal with." | 2-Jun |
| | Engaging in Hobby Activities or Past Times | Coping through leisure activities; motivations and purposes for engaging in hobbies (distraction, relaxation, creation, exercise, etc.) | Include any activity or past time mentioned with regards to either coping with life or coping in recovery from use; activities can involve elements of either social connection or isolation; can be adaptive or maladaptive | "I had one lady, she'd crochet when she'd feel like she'd get the urge because it's just about staying busy…"; "I rhinestone and I resin."; "Cooking or listening to music."; "Weight training." | 19-May |
| | Practicing Mindfulness and Internal Reflection | Coping through contemplative activities; motivations and purposes for practicing quiet | Include any activity that implies personal, individual thought, writing, feeling, etc. (as opposed to activities that are explicitly social or | "I just decided one day, 'You know what? I'm done. I'm so done," and I just prayed and prayed. That's why I'm so spiritual now."; "I like to journal a lot."; "Meditation." | 19-May |

| Subtheme | Code | Definition | Decision Rule | Example | First Occurrence |
|---|---|---|---|---|---|
| | | reflection (self-regulation, stoking spirituality or relationship with higher power, etc.) | distracting in nature); can be adaptive or maladaptive | | |
| | Managing Social Connectivity | Coping through fostering connection or seeking isolation; associated experiences (e.g., joining religious or recovery-oriented groups vs. disconnecting from loved ones) | Does not necessarily have to be an activity, but rather a way of living or being (i.e., choosing to be around others vs. to avoid others); can be adaptive or maladaptive; do not include broader mentions of social belonging (fall under Social Influence and Group Belonging) | "When I was in [treatment center] the only time I ever came outside of my room… was when the needle exchange would come…"; "basically I'm intertwined with my family most of the time…" | 19-May |
| Community Composition | Family Dynamics and Involvement | Family or close loved ones' approach to and understanding of substance use and recovery; influences of familial composition on individuals' health and wellbeing | Include any discussion around family interactions, family composition, and support exchanged between family members; descriptions of a family member's substance use would fall here (and may also fall under Transmission of Substance Use Knowledge: Interpersonal Role Models, if said family member's use exposes or influences other family members' behaviors with regards to use) | "[The increase in overdoses] is getting worse because, some people, kids actually sell to them or they buy it from the kids... Or if the parents don't get it from the kids, the kids get it from the parents." ; "…sometimes the family doesn't know [about a loved one's use] and that can look different from 'oh, I just don't wanna help...' but in their head, they probably just don't know how to help them," ; "When we talk about drug rehabilitation, we have to talk about all the dynamics that go along with what makes up a healthy family and what causes an individual to detour." | 19-May |
| | Social Influence and Group Belonging | Peer influences on substance use and recovery (i.e., peer | Membership in or exclusion from AA/NA groups, friend groups, neighbor groups, | "...you are being accepted by a whole group of people... accepted. Because if you don't smoke weed | 19-May |

| Subtheme | Code | Definition | Decision Rule | Example | First Occurrence |
|---|---|---|---|---|---|
| | | pressure vs. peer support; group inclusion vs. isolation) | etc.; do not include explicit mentions of seeking or avoiding social connection as a form of coping (falls under Managing Social Connectivity) | you can't hang around people who smoke weed. They don't wanna be bothered with you."; "When I was coming up, the older guys in the streets taught us that doing drugs was bad. If they found out you was doing drugs, they'd cut you off. Now the older guys in the streets are addicted themselves."; "Somebody that loves you will give you something that will nourish your body, [not hurt it]." | |
| | Neighborhood Relations and Community Cohesion | Dynamics present between members of the same neighborhood or community, including trust, cooperation, conflict resolution, communication, and engagement in shared activities; community culture, shared norms, and expectations | Descriptions of what characterizes the atmosphere of a neighborhood (i.e., presence and intensity of violent crime, finding community through sports, embodiment of the "Village Concept", availability and utilization of "third spaces," general trust (between neighbors, in resources, etc); overlap may occur between here and Impactful Life Experiences if the neighborhood environment is being described (as opposed to the family environment, which would fall under Family Dynamics and Involvement) | "My parents worked so the village raised us."; "Everybody distrusts everybody now…"; "Gang culture filled the void that was left when they took sports and all that stuff..."; "I've never seen a community come together like they do here. The way the community cares about each other, you can feel it." | 2-Jun |
| Psychological Experiences | Mental Health and Cognition | Mentions of mental health diagnosis, suicide, and general | Do not code negative emotions (i.e., stress), only professionally defined | "Most of the time it's the stress and depression that kills them for real… drugs will make them forget [mental | 2-Jun |

| Subtheme | Code | Definition | Decision Rule | Example | First Occurrence |
|---|---|---|---|---|---|
| | | mental processing (memory, attention, planning, etc.) | mental health conditions; do not code cognitive changes explicitly associated with substance use (this would be coded under Altering the Body and Mind) | illness]. When they come down they dead."; "I went over and did a community event… and I wanted to cry because out of the 60-something people that came over to my team, I could not hold a conversation with... not one of them." | |
| | Emotional Health and Resilience | Individuals' experience with feeling, recognizing, and balancing complex emotional states (e.g., numbness, guilt, anger, loneliness, fear, etc.) | Do not code coping mechanisms here (Coping Strategies); instead, code discussion around the feelings or emotional responses that precede or accompany them | "What bothers me is the guilt, the shame of realizing everything I've done to hurt the people I love"; "…I think all of us tip off the scale when something rolls… and we're like, 'I got to have something to help with that… it's like going underwater and you're just dog paddling and you get your little bit of air…" | 19-May |
| | Self-Concept and Awareness | Individuals' understanding and awareness of the self (i.e., self-esteem, aspects of identity, dignity, social comparison, locus of control) | Include statements that reveal individuals' perceptions around who they are, how they value themselves, who they want to be, etc. | "The fact of being homeless, your self-esteem is already compromised."; "The more that I educate myself, the more I feel better about myself. Lack of education, for me, made me feel less than…" | 2-Jun |
| | Sense of Safety | Feelings of safety, security, comfort, and familiarity as they relate to substance use and recovery | Can double code between here and Emotional Health and Resilience, provided that the emotional experience being described relates back to a deeper understanding of personal, neighborhood, or community safety (perceived threats can be | "It's so many people that are addicted to the sadness, the depression, the anger… people are scared to even feel a sense of happiness or love because they think it's gonna go away!"; "First time I picked up the vodka and thought, 'this is my buddy, this is my teddy bear, I ain't letting go,' ... and the fact there was no safety outward." | 13-Sep |

| Subtheme | Code | Definition | Decision Rule | Example | First Occurrence |
|---|---|---|---|---|---|
| | | | psychological or physical in nature) | | |
| Physiological Experiences | Seeking Homeostasis | Individuals' interactions with the embodied experience of using drugs, including pleasure-seeking and pain-avoidance behaviors | Behavioral changes associated with use (i.e., chasing the high, avoiding withdrawal, connection-seeking as an alternative to use ["addiction mimics connection"]); do not include mentions of bodily changes associated with use | "…some people get to the point where it be like, 'If I could just get past [detox], I would never ever put myself in the position where I'd be a slave to heroin,' you know? But you can't wake up and say, 'I'm gonna chill today,' you know? Your whole life is revolved around getting and using drugs." | 2-Jun |
| | Altering the Body and Mind | Mental or physical changes associated with substance use; the impacts of drugs or medications on bodily function and appearance | Bodily changes associated with use (e.g., blood pressure decrease, tolerance increase, side effects); do not include vague mentions of a drug's intended use (i.e., upper vs. downer) if no specific physiologic response is discussed; do not include behavioral changes associated with use | "Methadone… like I said, it's just a replacement. I see a lot of people who are losing their teeth, their bones are brittle, because of the long-term use of it."; "…your body will get adjusted to it, you know what I mean? And then you need more and more and more and more... until you overdose..." | 19-May |
| | Genetic Risk and Family History | Perceptions around individuals' hereditary predisposition to substance use | General discussion around genetic factors in substance use; do not include behavioral or physical changes associated with use | The reality of it is, addiction comes in many, many forms; it may be drugs, food sex. That's the addictive personality, and you can't fight that especially when it's in your DNA." | 13-Sep |
| Physical Health | Co-occurring Health Conditions or Injuries | Mentions of chronic pain, physical injury, or other existing health conditions when discussing | Include any diseases or health conditions existing before or in conjunction with substance use; do not include physical side effects | | |

| Subtheme | Code | Definition | Decision Rule | Example | First Occurrence |
|---|---|---|---|---|---|
| | | substance use and recovery-related experiences | | | |
| Faith and Spiritual Health | Religious Involvement and Spirituality | Mentions of personal faith and/or religious affiliation when discussing substance use and recovery-related experiences | Include all mentions of personal faith, spirituality, or organized religion (e.g., expressions of faith during recovery, engagement with faith-based services, discussion around local communities of faith, etc.) | "I go to church and I pray for the ones that I know are struggling with addiction, because it's hard… and I understand."; "That's why I like the churches, a lot of the churches are doing things [providing resources and services] for the right reasons…"; "If God can't fix our people... it's tough, you know?" | 2-Jun |
