# Supplementary material for: A community conversation process to establish resident and service provider perspectives on needs related to use and treatment of opioids and substances
Source: Front Public Health. 2026 Jan 27;13:1678130. doi: 10.3389/fpubh.2025.1678130 (PMC12886460; doi:10.3389/fpubh.2025.1678130)
Supplement: Supplementary file 1 [file Data_Sheet_1.zip › Appendix B, Fig. B.5 (Substance Use Behavior Survey).pdf]

Community Conversations on Overdoses in Richmond

What is your age? ____

What is your zip code? _______________________________________

Which most closely describes your race/ethnicity?

- ☐ American Indigenous/Alaskan Native
- ☐ Asian
- ☐ Black/African American
- ☐ Hispanic/Latinx
- ☐ Native Hawaiian/Pacific Islander
- ☐ White
- ☐ Not listed. My race/ethnicity is:

  ____

- ☐ Prefer not to say.

Which most closely describes your gender?

- ☐ Woman
- ☐ Man
- ☐ Transgender Woman
- ☐ Transgender Man
- ☐ Non-Binary
- ☐ Agender/I don't identify with any gender.
- ☐ Not listed. My gender is:

  ____

- ☐ Prefer not to say.

At what age did you *start* using each of the following substances?

	**Age**	**I have never used this substance**	**I don't know**	**I choose not to answer**
Tobacco (e.g., cigarettes, e-cigarettes, cigars)				
Alcohol				
Cannabis (marijuana, hashish, THC liquid)				
Cocaine (powder, crack)				
Amphetamines/Methamphetamines (e.g., MDMA, Molly, Ecstasy, Dexedrine, Ritalin, Adderall)				
Hallucinogens (e.g., LSD, magic mushrooms, psilocybin, peyote, Ketamine, Dextromethorphan)				
Opiates (e.g., heroin, morphine, codeine, opium, kratom)				
Prescription opioids (e.g., fentanyl, tramadol, oxycodone/OxyContin/Percocet, methadone, hydrocodone/Vicodin, hydromorphone/Dilaudid, meperidine/Demerol, buprenorphine)				
Other substance, please specify:				

How many times have you used the following substances *in your life*?

	0 times	1-9 times	10-99 times	100 or more times	I choose not to answer
Tobacco (e.g., cigarettes, e-cigarettes, cigars)					
Alcohol					
Cannabis (marijuana, weed, hashish, THC liquid)					
Cocaine (powder, crack)					
Amphetamines/Methamphetamines (e.g., MDMA, Molly, Ecstasy, Dexedrine, Ritalin, Adderall)					
Hallucinogens (e.g., LSD, magic mushrooms, psilocybin, peyote, Ketamine, Dextromethorphan)					
Opiates (e.g., heroin, morphine, codeine, opium, kratom)					
Prescription opioids (e.g., fentanyl, tramadol, oxycodone/OxyContin/Percocet, methadone, hydrocodone/Vicodin, hydromorphone/Dilaudid, meperidine/Demerol, buprenorphine)					
Other substance, please specify:					

When was the *last time* you used any of the following substances for any reason?

	I have never used this substance	In the last 3 months	4-24 months ago	More than 2 years ago	I choose not to answer
Tobacco (e.g., cigarettes, e-cigarettes, cigars)					
Alcohol					
Cannabis (marijuana, hashish, THC liquid)					
Cocaine (powder, crack)					
Amphetamines/Methamphetamines (e.g., MDMA, Molly, Ecstasy, Dexedrine, Ritalin, Adderall)					
Hallucinogens (e.g., LSD, magic mushrooms, psilocybin, peyote, Ketamine, Dextromethorphan)					
Opiates (e.g., heroin, morphine, codeine, opium, kratom)					
Prescription opioids (e.g., fentanyl, tramadol, oxycodone/OxyContin/Percocet, methadone, hydrocodone/Vicodin, hydromorphone/ Dilaudid, meperidine/Demerol, buprenorphine)					
Other substance, please specify:					

Has a relative, a friend, a doctor, or another health worker been concerned about your use or suggested you cut down due to your use of the following substances?

	I have never used this substance	Yes	No	I don't know	I choose not to answer
Tobacco (e.g., cigarettes, e-cigarettes, cigars)					
Alcohol					
Cannabis (marijuana, hashish, THC liquid)					
Cocaine (powder, crack)					
Amphetamines/Methamphetamines (e.g., MDMA, Molly, Ecstasy, Dexedrine, Ritalin, Adderall)					
Hallucinogens (e.g., LSD, magic mushrooms, psilocybin, peyote, Ketamine, Dextromethorphan)					
Opiates (e.g., heroin, morphine, codeine, opium, kratom)					
Prescription opioids (e.g., fentanyl, tramadol, oxycodone/OxyContin/Percocet, methadone, hydrocodone/Vicodin, hydromorphone/Dilaudid, meperidine/Demerol, buprenorphine)					
Other substance, please specify:					

Have you *ever tried to quit* using any of the following substances for more than 24 hours?

	I have never used this substance	Yes	No	I don't know	I choose not to answer
Tobacco (e.g., cigarettes, e-cigarettes, cigars)					
Alcohol					
Cannabis (marijuana, hashish, THC liquid)					
Cocaine (powder, crack)					
Amphetamines/Methamphetamines (e.g., MDMA, Molly, Ecstasy, Dexedrine, Ritalin, Adderall)					
Hallucinogens (e.g., LSD, magic mushrooms, psilocybin, peyote, Ketamine, Dextromethorphan)					
Opiates (e.g., heroin, morphine, codeine, opium, kratom)					
Prescription opioids (e.g., fentanyl, tramadol, oxycodone/OxyContin/Percocet, methadone, hydrocodone/Vicodin, hydromorphone/Dilaudid, meperidine/Demerol, buprenorphine)					
Other substance, please specify:					
