# Supplementary material for: A community conversation process to establish resident and service provider perspectives on needs related to use and treatment of opioids and substances
Source: Front Public Health. 2026 Jan 27;13:1678130. doi: 10.3389/fpubh.2025.1678130 (PMC12886460; doi:10.3389/fpubh.2025.1678130)
Supplement: Supplementary file 1 [file Data_Sheet_1.zip › Appendix B, Fig. B.2 (CHW Program Brochure).pdf]

NEED HELP?

Being healthy means having what you need in every part of your life.

A Community Health Worker can help you connect to resources for:

- Food
- Employment
- Transportation
- Housing
- Insurance
- Healthcare
- Social Services
- Car seats & much more...

CHWs support COVID-19 response efforts.

MORE INFO

Scan the QR code for more information on clinic resources and hours.

Richmond City Health District (Main location)
804-482-5500
400 East Cary St
Front entrance is located on Main Street

rhhd.gov/rc

Follow each Resource Center on Facebook

RHHD RESOURCE CENTERS
Bridge to a Healthy Community

Community HEALTH WORKER Program

DON'T DELAY, CONTACT US TODAY!

RICHMOND CITY HEALTH DISTRICT | **HENRICO COUNTY** HEALTH DISTRICT

WHO ARE THE COMMUNITY HEALTH WORKERS?

Community Health Workers are community leaders who can help connect you to resources in the community and advocate for your voice to be heard.

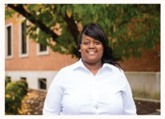

Stephanie Carrington, CCHW Sr
CCHW Senior for the East End
Office: (804) 371-0433
Cell: (804) 506-3430
Stephanie.Carrington@vdh.virginia.gov
Facebook: Creighton Resource Center

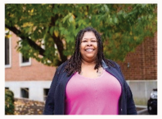

Patrice Shelton, CCHW Sr
Hillside CHW
Office: (804) 230-7740
Cell: (804) 404-5166
Patrice.Shelton@vdh.virginia.gov
Facebook: Hillside Resource Center

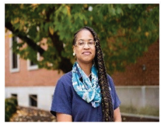

Keandra Holloway, CCHW
Fairfield CHW
Office: (804) 786-4099
Cell: (804) 362-8164
Keandra.Holloway@vdh.virginia.gov
Facebook: Fairfield Resource Center

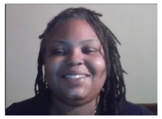

Tanya Simms, CCHW
Gilpin CHW
Office: (804) 219-9126
Cell: (804) 223-0437
Tanya.Simms@vdh.virginia.gov
Facebook: Gilpin Resource Center

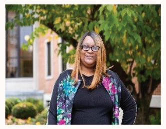

Ivy Bell, CCHW
VCU Health Hub at 25th
Office: 804-628-6403
Cell: (804) 506-3247
Ivy.Bell@vdh.virginia.gov

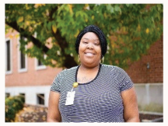

Alante Cannon, CCHW
Mosby CHW
Office: (804) 786-0204
Cell: (804) 424-0391
Alante.Cannon@vdh.virginia.gov
Facebook: Mosby Resource Center

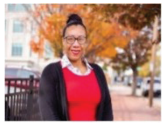

Yovaldi Lamoutt, CCHW
Southwood CHW
Cell: (804) 584-6439
Yovaldi.Lamoutt@vdh.virginia.gov
Facebook: Southwood Resource Center

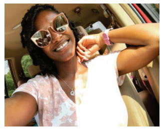

Quawneisha Peoples, CCHW
Whitcomb CHW
Office: (804) 786-0555
Cell: (804) 914-2781
Quawneisha.Peoples@vdh.virginia.gov
Facebook: Whitcomb Resource Center

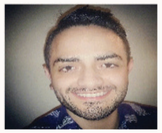

Mario Martinez, CHW
Southwood CHW
Cell: (804) 517-3337
Mario.Martinez@vdh.virginia.gov

HOW CAN WE HELP?

Community Health Workers strengthen their communities by helping residents connect to resources, learn about health, and speak out for positive change.

- We offer door-to-door outreach in the community and attend outreach events to **RELATE** to the community at the library, local churches and businesses, etc. where we provide blood pressure and blood sugar screenings and give away free condoms.
- We provide one-on-one **EDUCATION** and large group informational sessions such as REVIVE Narcan training and Chronic Disease Self Management workshop.
- We help you **NAVIGATE** medical and social resources such as finding a primary care doctor, applying for health insurance, access to food, housing, transportation, employment, etc.
- We ensure the community has a voice that influences policy changes, **ADVOCATING** for improvements you want to see.
