# Supplementary material for: A community conversation process to establish resident and service provider perspectives on needs related to use and treatment of opioids and substances
Source: Front Public Health. 2026 Jan 27;13:1678130. doi: 10.3389/fpubh.2025.1678130 (PMC12886460; doi:10.3389/fpubh.2025.1678130)
Supplement: Supplementary file 2 [file Data_Sheet_2.pdf]

Appendix D: Additional Themes

Conversation Findings: “What factors are contributing to the increase in overdoses in Richmond?”

Diversity in Substance Use Narratives

Range of “Drug” Definitions. Participant definitions of what constituted a “drug” varied. For example, four participants defined a substance as a “drug,” based on the presence and strength of its direct physiological effects. In contrast, three others focused on the manner in which it is obtained, considering any substance to be a drug if not formally prescribed by a qualified health professional: *“If it ain’t prescribed to you and you ain’t supposed to take it, then it’s definitely a drug.”* Discussion also revealed a variety of colloquial names for substances that may be community- and region-specific: *“In the country… we call crack ‘boy’ and soft ‘girl,’ but up here [in the city] they call hard ‘girl’ and boy ‘heroin.’”* Participants thought this inconsistency could reflect wider public confusion related either to drug potency or to which specific substances could lead to overdose.

Social Determinants of Substance Use. Socioeconomic factors (e.g., finances, housing, childcare access, and transportation) were cited by 12 participants in discussion around substance use. Participants highlighted these factors’ complex influence on clinical treatment access and community resource engagement.

As important lenses through which individuals engage with the world, gender and age were also thought to influence substance use. Five participants described how an individual’s use may differ based on gender-related experiences (e.g., societal treatment, roles, responsibilities, and expectations). Speaking of their daughter, one participant suggested: “*Some women do it because they have low self-esteem… feeling unloved makes them do it, feeling depressed.”* Additionally, four participants perceived age-related differences in population-level use patterns, suggesting that youth ages 16-25 are more significantly affected by substance use than other age groups. However, this view was not consistently held; four other participants felt that all age groups are equally affected.

Diversity in Perceptions on Recovery. Participants acknowledged many “paths” to successful recovery, but perspectives differed on the roles of individual agency and external support in initiating it. For example, 10 participants described the decision to seek recovery as deeply personal, emphasizing that an individual must reach a point of internal readiness to begin: *“You can pray, you can throw holy water on them, they can go to 50 treatment centers and it’s not going to help until that person gets sick and tired. That’s the bottom line.”* Conversely, 10 participants emphasized the importance of external supports in motivating individuals to reach internal readiness and maintain commitment: *“...it could be a friend or family member. You gotta have somebody there to push you… it just takes that one person.”* Five of these participants identified an individual’s social environment across all stages of recovery as a key predictor of success, cautioning that recovery could unexpectedly turn toward use without the presence of consistent, appropriate supports: *“I feel like support in later recovery is a lot*

more important because so many people when you first come in – they're so supportive and they surround you and they're really encouraging and they believe in you, but then you get to this stage… with what's next… that's when you go back to old behaviors."

Conversations also highlighted disagreement around the effectiveness of harm reduction strategies used to promote recovery in Richmond. Thirteen participants offered positive feedback about these strategies' (e.g., naloxone distribution, clean needle programming) ability to prevent death among those engaged in use. One participant described harm reduction as an important step in reaching and protecting people: *"[It's about] meeting folks where they are."* However, four of those who supported harm reduction principles also expressed disappointment that the current harm reduction strategy was almost exclusively limited to naloxone distribution. One participant, and service provider, explained: *"I was at [a local community event] … and the thing that upset me was there were maybe 15 vendors and four of us were giving out Narcan. And it's like, this is how we approach this problem. All we do is flood with Narcan, and we don't think about prevention, like there's no… there's no more than that. We, you know, check the box – we done it."* Further, these participants cautioned that, as a tertiary prevention strategy, harm reduction can only make up one part of a comprehensive approach to substance use prevention: *"Harm reduction is not going to be effective if you're not addressing the trauma [underlying substance use] ... it's gotta be part of a larger picture."*

Participants expressed mixed opinions regarding the use of medication-assisted treatment (MAT) for recovery. These opinions appeared to vary between individuals based on their personal experience receiving MAT as a mode of treatment, with one notable exception. Speaking observationally, seven participants expressed the belief that MAT (e.g., suboxone and methadone) contributes to substance use, stating that such treatments are "opioid substitutes." One participant believed that many people taking methadone through a legitimate medical treatment plan had not ceased consumption of street drugs: *"Methadone, it's just a replacement… I see a lot of people who are losing their teeth, their bones are brittle, because of the long-term use of it… I think most of the people who take methadone do street drugs."* However, another participant likened MAT to "keeping yourself sick," attesting that the ability to sell their own MAT doses funded their continued substance use. In contrast, four participants shared positive personal experience with methadone and suboxone in preventing fatal overdose (*"Methadone will give you your life back—your quality of life back… There are a lot of people, they all say, 'Oh, you just changing one drug for another,' and I say, 'Look, yeah I am and just be glad that I am, because I ain't trying to be dead.'"*). In response to negative perceptions expressed by other participants, these participants conceded that methadone was not meant as a "forever" prescription but maintained that it was necessary to begin finding new balance, structure, and stability.

Coping with Impactful Life Events and Mental Health Experiences

This theme represents the personal life experiences that participants identified as having psychological implications related to substance use. Participants often discussed negative life events or challenges preceding substance use as well as engagement in substance use as a coping strategy to address unresolved mental health issues.

Life Events Influencing Substance Use. The most frequently identified precursor to substance use was emotional pain, trauma, and negative mental health experiences that accumulated across the life course: *"When I was coming up, I had a lot of childhood trauma… unhealthy environment at home… drinking and drugs. That had an effect on me at an early age. Now the more I educate myself, the more I feel better about myself. Lack of education made me feel less than, and I wanted to mask that feeling. The drugs gave me freedom from that. When it wore off, I still had to face… whatever I created when I was under the influence. That made me go deeper, now I got to use more… the bigger the level, the bigger the devil. The bigger the level of pain that you experience within you… the more sh-- you've been through… I'm trying to cover that hurt up."* Eight participants discussed other interpersonal experiences that impacted substance use, including domestic violence, being bullied as a child, adverse experiences in foster care, being sex trafficked, and general relational trauma.

Ten participants expressed that the intense emotional pain of losing a loved one—often an essential social support—facilitated their substance use. For example, one participant discussed substance use as a form of suicidal behavior following death of a spouse: *"My husband overdosed. He passed away and then I started using… because I thought if I got the right stuff, I could kill myself."* However, loss produced different results for others; one participant reported that losing a loved one to overdose motivated her to stop using entirely, describing the experience as a "wake-up call."

Substance Use as a Coping Strategy to Address Mental Health Issues. Nineteen participants discussed substance use as a coping mechanism to address undiagnosed mental illness, emotional imbalance, or untreated physical pain: "*I know someone whose partner got shot and almost lost their life. I know there are some feelings, like when you're close to losing your livelihood, you're dealing with some feelings or depression, or you're scared, or you may be paranoid, and instead of seeking help with that he decided to start medicating on the street drugs... to cope with the feelings. That goes with that stigma, like, "I gotta pretend that I'm okay," and… the romance behind, like, "it's okay if I can take these drugs, but it's not okay if I talk about my feelings.*"

Three participants with personal substance use experiences also attributed their behaviors to the sense of reliability, safety, and security associated with the escape and euphoria of substance use: *"First time I picked up the vodka and thought, 'This is my buddy. This is my teddy bear. I ain't letting go.' … There was no safety outward."* Another participant, and service provider, elaborated by suggesting that unresolved mental or emotional health concerns can create a cycle of familiar negativity, often characterized by continued use: *"It's so many people that are addicted to the sadness, the depression, the anger… people are scared to even feel a sense of happiness or love because they think it's gonna go away!"*

Conversation Findings: What resources or support services are missing that could be implemented in the community?

Consistent Wraparound Resource Support

This theme focuses on the types and characteristics of social supports described by participants. In general, participants indicated the need for high quality, affordable, personalized, and flexible resource support that acknowledges the unique challenges of people engaged in substance use, those directly affected by addiction/substance use disorder, or the people supporting these individuals (e.g., resource support staff or family members). Two settings were recognized to benefit from the inclusion of coordinated comprehensive support: corrections and health care. Additionally, participants noted the need for consistently available resources that would offer social support across a variety of areas that often contribute to or are exacerbated by substance use.

<u>Social Support Resource Needs</u>. Housing and employment were identified as interconnected, high-priority resource needs that also uniquely affect people engaged in substance use. First, nine participants noted the on-going issue of limited bed space in government-funded shelters. Second, there were perceived issues in accessing affordable housing due to institutional policies that prioritized some groups (e.g., women and children) while also offering equal access to all applicants, regardless of Richmond city residency. Consequently, many participants found it challenging to secure affordable housing against a growing pool of applicants where certain groups were treated preferentially. As a result, participants indicated that the supports available to access the already limited supply of affordable housing units were similarly limited.

Fifteen participants also shared the unique challenges of accessing affordable housing while managing substance use or after incarceration. For example, participants who have experienced homelessness reported engaging in substance use to cope with the experience of being homeless. However, they also expressed frustration that many housing communities required clean and sober living upon arrival, which may not be immediately possible. A need for more housing options was discussed, including transitional housing options that offer space across substance use circumstance, affordable housing options free of substances for people in recovery, and day support shelters which offer supports for people who are not housed (e.g., on a wait list) with the ability to access beyond working hours. Participants acknowledged the presence of such options but identified challenges in appropriately matching to the right housing community given personal circumstances.

Second, participants identified the overlap between housing and employment and described how the presence of both could lead to improved outcomes. Housing offered a means to support basic needs (e.g., hygiene, safety, food, address to include in job applications), which improved the likelihood of obtaining employment. In turn, employment offered income and stability, which were considered to lead to sustained housing. Additionally, maintaining employment was considered to contribute to self-confidence and self-esteem (*"You need the routine job... the routine of social interactions with people outside of the recovery house. Because when... the only people that you are living with are going through addiction, sometimes that can make you feel isolated as well—like you aren't really part of the world, you are just part of this world [of substance use and recovery]"*). In the absence of consistent housing and employment, participants supported resources such as day shelters to address basic needs while

seeking permanent solutions ("*The fact of being homeless, your self-esteem is already compromised. You already feel some type of way about yourself, so being that we don't have a lot of shelters– we don't have any like, there's [unintelligible program name] … It's a day support shelter. …You can go in there and literally walk in the door and say, 'Hey, I don't have my ID.' Then they'll link you up with a case worker, and she comes out there like twice a week. You get your ID. Everything. They got, like services, the services there… anybody can go there and take showers, wash your clothes. … Another day you come over, getting together and everything like take a shower, I can shave. So, I'm feeling myself. But if you want me to go get a job when I didn't take a shower in like four days...*").

Ten participants identified unique challenges for formerly incarcerated individuals in seeking employment, noting that this set of specific stressors required additional support. They noted that many formerly incarcerated individuals lack the documentation (e.g., driver's license, birth certificate) to successfully complete applications or onboarding processes for employment. Housing applications routinely require the same materials. Previously incarcerated individuals also discussed employer hesitancy to hire an applicant with a criminal record. Additionally, one participant expressed frustration with the practice of resource support elimination after meeting milestones such as securing work and housing, even if an individual felt unprepared for such a change. As a result, the following resource needs were identified: (1) access to workforce skills training; (2) rapid access to important documentation for job and housing applications; (3) access to second-chance employment opportunities; (4) access to second chance finance opportunities (e.g., loans and bank accounts); (5) job search support; (6) personal finance and money management skills; (7) support for maintaining well-being (e.g., substance use peer recovery support groups, wraparound services for the family unit); and (8) preparation prior to transitions through major milestones (e.g., leaving prison) alongside related reduction/loss of resources, benefits, and oversight. Five participants advocated for the availability of services like these in a single, accessible location: "*[The community needs] a one-stop shop. Like the building that we in right now, right? Imagine if that building had everything in there—had a rep. from social services in there, had somebody from the health department in there, had somebody from every facet. So, it's a one-stop shop and you ain't got to run around. You know? I'm saying it's all right here, even with the government and workforce development…*"

<u>Health-Related Resource Needs</u>. Participants who received services from resource organizations or those who were resource providers identified several common issues. First, the quality of resource support varied widely across organizations. Many were described as providing low quality services or having little oversight, which led to easy substance access while in or around the treatment center ("*I was just with somebody today and she said, 'I don't want to go to [Name of Treatment Center], because they have drugs right across the street.'*"). Eleven participants discussed situations where they did not perceive the organization to be committed to the wellness of the client ("*The house wasn't making them do anything for their sobriety. It was just kind of like one of those houses that's just for the money type thing… in and out, in and out, in and out type thing.*"). Second, six participants expressed frustration with treatment duration limits placed by insurance company

policies. One service provider remarked: "*So you know recently, probably in the past maybe six months increasingly, I have submitted authorizations to Managed Care Organizations and I had one send back to me… that I can provide four days of treatment, 3 hours a day, for 30 days. I said, '30 days?' They take 30 days to get on it… to get hooked*." Participants did not consider such policies conducive to effectively supporting people faced with addiction or substance use disorder associated with other unresolved mental health conditions ("*I don't think that the 30-day treatment facilities are really a solution. They're very expensive. And after 30 days you're just done. Like, I don't feel like a problem that I spent years creating I'm going to be able to heal in 30 days*."). Third, services were not coordinated across organizations, leading to gaps in support and negative outcomes ("*Once they get out of [hospital name], y'all put him in a treatment center... or put him somewhere that he can have the counseling services, and phone calls, and people all around to try to assist him in that situation. All they do is, when he left from [hospital name], they threw him in a hotel, he called his drug man who went and got him some fentanyl, OD'd and died."*). One participant reported unsuccessful attempts to enroll a loved one in a local substance use treatment facility: *"It just seems like all of it is a Catch 22. It's, 'you don't qualify for the services,' or 'you do qualify for the services,' but they can't help you."* Consequently, several resource needs connected with the health care setting were identified: (1) affordable residential treatment facilities, (2) medical support during detoxification, (3) addiction treatment as an alternative to incarceration, (4) continued post-treatment "aftercare", (5) mental health care, (6) continued funding and professional development for resource providers, and (7) access to treatment intake and resource support opportunities outside typical office settings (e.g., community events).

Overall, participants were mixed on the resources available to address substance use and overdose. While 14 expressed varying degrees of hopelessness or apathy for the future (*"You know what? We just need to admit that society has… society has failed, especially low-income communities."*), 10 expressed some notion of continued hope *("It probably gotta be grassroots, you know? You take one section, mold it, then you go on to the next and then slowly but surely it'll catch on. You'll end up having a whole city behind you because you see that changes are being made…").* One participant in particular acknowledged the community's existing capacity for mutual support and solidarity: *"I've never seen a community come together like they do here. The way the community cares about each other… you can feel it."*
